# Stakeholder-driven transformative adaptation is needed for climate-smart nutrition security in sub-Saharan Africa

**DOI:** 10.1038/s43016-023-00901-y

**Published:** 2024-01-02

**Authors:** Stewart Jennings, Andrew Challinor, Pete Smith, Jennie I. Macdiarmid, Edward Pope, Sarah Chapman, Catherine Bradshaw, Heather Clark, Sylvia Vetter, Nuala Fitton, Richard King, Sithembile Mwamakamba, Tshilidzi Madzivhandila, Ian Mashingaidze, Christian Chomba, Masiye Nawiko, Bonani Nyhodo, Ndumiso Mazibuko, Precious Yeki, Pamela Kuwali, Alfred Kambwiri, Vivian Kazi, Agatha Kiama, Abel Songole, Helen Coskeran, Claire Quinn, Susannah Sallu, Andrew Dougill, Stephen Whitfield, Bill Kunin, Nalishebo Meebelo, Andrew Jamali, Dhaquirs Kantande, Prosper Makundi, Winfred Mbungu, Frank Kayula, Sue Walker, Sibongile Zimba, Joseph Hubert Galani Yamdeu, Ndashe Kapulu, Marcelo Valadares Galdos, Samuel Eze, Hemant Tripathi, Steven Sait, Stefan Kepinski, Emmanuel Likoya, Henry Greathead, Harriet Elizabeth Smith, Marcelin Tonye Mahop, Helen Harwatt, Maliha Muzammil, Graham Horgan, Tim Benton

**Affiliations:** 1 Institute for Climate and Atmospheric Science, School of Earth and Environment, University of Leeds, Leeds, United Kingdom; 2 Institute of Biological and Environmental Sciences, University of Aberdeen, Aberdeen, United Kingdom; 3 The Rowett Institute, School of Medicine, Medical Sciences and Nutrition, University of Aberdeen, Aberdeen, United Kingdom; 4 Hadley Centre, Met Office, Exeter, United Kingdom; 5 The Global Systems Institute, University of Exeter, Exeter, United Kingdom; 6 Institute of Applied Health Sciences, School of Medicine, Medical Sciences and Nutrition, University of Aberdeen, Aberdeen, United Kingdom; 7 Chatham House, The Royal Institute of International Affairs, London, United Kingdom; 8 Food, Agriculture and Natural Resources Policy Analysis Network, Pretoria, South Africa; 9 Agricultural Consultative Forum, Lusaka, Zambia; 10 National Agricultural Marketing Council, Pretoria, South Africa; 11 Civil Society Agriculture Network, Lilongwe, Malawi; 12 Economic and Social Research Foundation, Dar es Salaam, Tanzania; 13 School of Biology, Faculty of Biological Sciences, University of Leeds, Leeds, United Kingdom; 14 Sustainability Research Institute, School of Earth and Environment, University of Leeds, Leeds, United Kingdom; 15 Regional Network of Agricultural Policy Research Institutes, Lusaka, Zambia; 16 Malawi National Planning Commission, Lilongwe, Malawi; 17 Concern Worldwide, Lilongwe, Malawi; 18 Environmental Management Unit, Ministry of Agriculture, Dodoma, Tanzania; 19 Sokoine University of Agriculture, Morogoro, Tanzania; 20 Kaypro Research Institute, Lusaka, Zambia; 21 Agricultural Research Council, Pretoria, South Africa; 22 University of the Free State, Bloemfontein, South Africa; 23 Lilongwe University of Agriculture and Natural Resources, Lilongwe, Malawi; 24 School of Food Science and Nutrition, University of Leeds, Leeds, United Kingdom; 25 Section of Natural and Applied Sciences, School of Psychology and Life Sciences, Canterbury Christ Church University, Canterbury, United Kingdom; 26 Sustainable Soils and Crops, Rothamsted Research, Harpenden, United Kingdom; 27 Department of Agriculture and Environment, Harper Adams University, Newport, United Kingdom; 28 UN Environment Programme, World Conservation Monitoring Centre (UNEP-WCMC), Cambridge, United Kingdom; 29 USAID West Africa Biodiversity and Low Emissions Development (WABiLED) Programme, Accra, Ghana; 30 Biomathematics and Statistics Scotland, Aberdeen, United Kingdom

**Keywords:** Climate-change impacts, Climate-change mitigation, Climate-change policy, Developing world, Agriculture

## Abstract

Improving nutrition security in sub-Saharan Africa under increasing climate risks and population growth requires a strong and contextualized evidence base. Yet, to date, few studies have assessed climate-smart agriculture and nutrition security simultaneously. Here we use an integrated assessment framework (iFEED) to explore stakeholder-driven scenarios of food system transformation towards climate-smart nutrition security in Malawi, South Africa, Tanzania and Zambia. iFEED translates climate–food–emissions modelling into policy-relevant information using model output implication statements. Results show that diversifying agricultural production towards more micronutrient-rich foods is necessary to achieve an adequate population-level nutrient supply by mid-century. Agricultural areas must expand unless unprecedented rapid yield improvements are achieved. While these transformations are challenging to accomplish and often associated with increased greenhouse gas emissions, the alternative for a nutrition-secure future is to rely increasingly on imports, which would outsource emissions and be economically and politically challenging given the large import increases required.

## Abstract (web summary)

Effective solutions for food systems transformation must be designed in a participatory way. This study illustrates the application of an integrated assessment framework to explore stakeholder-driven scenarios towards climate-smart nutrition security in Malawi, South Africa, Tanzania and Zambia.

## Main

Achieving an adequate supply of energy and nutrients to meet population dietary needs under climate change requires policy decisions made in the face of high uncertainty across multiple components of complex socio-environmental systems^1^. This challenge is particularly urgent in sub-Saharan Africa (SSA), where climate change could put millions more people at risk of food and nutrition insecurity by mid-century^2^. At country scales and above, policies need holistic evidence if adaptation to climate change is to avoid being siloed in different government departments^3^.

The evidence available to inform agricultural policies that are resilient to climate change and can supply sufficient energy and nutrients to a population can be grouped into two broad areas: climate-smart approaches and transformative adaptation. Climate-smart agriculture (CSA), and more broadly climate-smart food systems^4^, consider the need for increased productivity and adaptation to climate change and the potential for mitigation—that is reducing the greenhouse gas (GHG) emissions resulting from adaptations. Transformative adaptation consists of structural changes to shift away from undesirable food system trajectories, rather than incremental coping mechanisms that characterize most policy approaches to climate and nutrition^5,6^.

The methods used for assessing the efficacy of transformative adaptation and CSA strategies are varied, ranging from modelling-based approaches that quantify uncertainties in climate change impacts to stakeholder-driven approaches that examine capacities and vulnerabilities^1^. Integrated Assessment Models (IAMs) have been used to assess food system options and outcomes under different future conditions, including land-use change, and environmental and economic impacts^7^. Uncertainties in possible food system futures can be explored using IAM scenario analysis^8–12^—for example, the widely used Shared Socioeconomic Pathways focus on energy, land use and mitigation^13^. Other analyses have explored scenarios of food production and consumption given climate change and policy decisions^14^ and the health implications of different future diets^7,15^. Few large-scale studies integrate nutrition or the importance of the trade-offs involved in achieving CSA, for example, optimizing water use and GHG productivity in rice systems^16^, or trade-offs between biodiversity and crop productivity^17^.

Nutrition and nutrient adequacy to meet population-level dietary needs have yet to be assessed within an integrated CSA framework, risking sub-optimal adaptation from both health and environmental perspectives^18^. Equally, studies of transformative adaptation have shown that historical food system transitions can have sub-optimal nutritional and environmental outcomes^19^, with most studies focussing on a small number of cereal crops and food production^20,21^. This suggests that the evidence on which current adaptation strategies are based is insufficient for achieving the changes needed for sustainable, climate-smart nutrient supply.

Inclusive approaches to integrated assessment—involving stakeholders at every stage of the process—are critical for informing country-specific policy processes. We use an integrated assessment framework—the integrated Future Estimator for Emissions and Diets (iFEED^22^)—to combine climate–food–emissions modelling with stakeholder and academic expertise, assessing both CSA and food and nutrient supplies and bridging the gap between modelling and national-scale policy-relevant outputs^23,24^. We assess the adequacy of energy and nutrient supplies to meet dietary requirements at a population level (hereafter referred to as population-level nutrition security). We describe results for four focal SSA countries: Malawi, South Africa, Tanzania and Zambia.

Our approach has three major steps, corresponding to the headings that follow. We first co-develop different possible future scenarios with stakeholders, designed to explore as broad a range of context-specific food system futures as possible. Scenarios use integrated modelling to analyse nutrition security and climate smartness. We then compare results across scenarios and countries, resulting in conclusions that are less sensitive to underlying assumptions and therefore more robust. Finally, we assess the policy implications of the findings.

## Climate-smart, nutrition-secure scenario assessment

We explore stakeholder-driven scenarios that assess how climate-smart nutrition security can be achieved by mid-century given population growth and increasing climate volatility in each focal country. Modelling of climate and land-use change and resulting impacts on domestic food production and agricultural GHG emissions are supported by comprehensive uncertainty reporting. The model results provide the basis for: (1) an analysis of how domestic production and trade interact in changing population-level nutrition security; (2) a diverse array of implication statements, including environmental and social implications of the results. This provides information for assessing nutrition security and CSA for each scenario. We then compare scenarios to identify robust commonalities that lead to preferred CSA and nutritional outcomes and lastly point to policy implications.

**Fig. 1:**
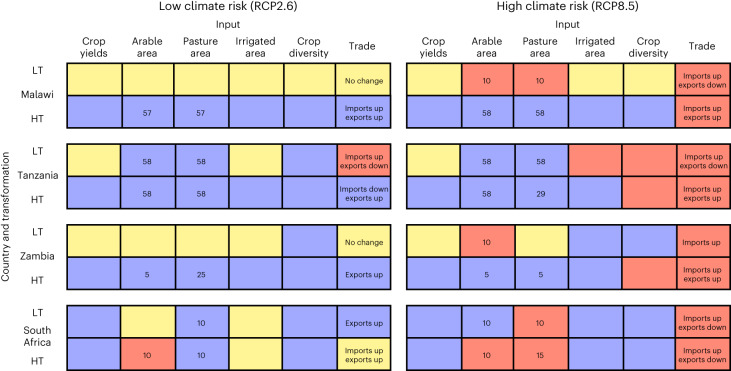
Scenario inputs to iFEED from stakeholder engagement. LT = low-transformation scenarios (low policy efficacy in Malawi; low market connectivity in Zambia; low technological development in Tanzania; low land reform in South Africa) and HT = high transformation. RCP2.6 = low climate risk. RCP8.5 = high climate risk. For arable area and pasture area, numbers given are percentage changes to land areas relative to a 1990–2010 baseline. The Malawi and Tanzania scenarios that feature agricultural area expansion use up all available land in mid-century (protected areas, urban areas and forests excluded), other than the Tanzania HT-RCP8.5 scenario where the livestock expansion was described by stakeholders to be smaller. Optimization to maximize domestic crop production was assumed in HT scenarios in Malawi, Tanzania and Zambia. Increasing crop diversity refers to maize areas decreasing and other crop areas expanding; decreasing crop diversity refers to maize areas increasing and other crop areas contracting. For each box: blue = increase; amber = no change; red = decrease. Note that the trade column refers to changes in imports/exports in the stakeholder-designed trade vignette, with the colour referring to increases/decreases in trade surplus, for example, whether imports increase more than exports.

Stakeholders created a 2 × 2 scenario matrix for each country during participatory scenario workshops. The stakeholders were representatives of government, academia, civil society and the agriculture sector, representing a broad a range of food system expertise (Supplementary Table 6 provides stakeholder details). How adaptation to climate change is implemented in each scenario is directly informed by stakeholders, ranging from the incremental (consisting of changes in planting dates and currently existing crop varieties) to the transformative (where different crops are grown in new locations to maximize production). Contrasting trade vignettes explore how business as usual or changes to trade impact nutrition security given domestic policy decisions. Figure 1 summarizes the outcomes of this process, including the assumptions around land use, crop yields, diversification and trade that underpin each scenario.

In all countries, the level of climate risk was selected by stakeholders as one of two critical uncertainties of food system futures. Low climate risk was characterized by 18 bias-corrected Coupled Model Intercomparison Project Phase 5 (CMIP5) climate models under the Representative Concentration Pathway RCP2.6 and high climate risk by RCP8.5^25^. Whereas extreme climate events (such as droughts, floods and record-breaking high temperatures) feature in projections for all countries and scenarios, they do not directly affect average future levels of nutrition security or CSA outcomes. However, resilience to extremes, as achieved through crop diversification, was found to have some nuanced implications for nutrition security (‘Micronutrient-rich, productive crops for nutrition security’ section). Extreme events were also important to stakeholders, and their implications are explored in Supplementary Text 1.

Stakeholders defined the second critical uncertainty around the agricultural transformative changes relevant to their country. These were the effectiveness of policy implementation (Malawi), the extent of land reform (South Africa), the extent of technological transformation (Tanzania) and the degree of market connectivity and functionality (Zambia). We refer to these simply as high-transformation (HT) or low-transformation (LT) scenarios. The result is four scenarios per country, comprising HT/LT × high/low climate risk.

Compared with the South Africa scenarios, Malawi, Tanzania and Zambia scenarios explore a larger range of adaptation options from small incremental changes to the transformative, and therefore we focus on these comparisons in the ‘Agricultural transformations for nutrition security’ section to assess the potential of such changes for improving nutrition security. HT scenarios for these three countries were associated with the largest changes in agricultural systems, characterized as having a continuation of historical yield trends, crop switching to maximize production and expansion of agricultural area and irrigation. These stakeholder-led scenario characterizations mean that yields generally increase in HT scenarios. LT scenarios in these three countries were characterized as more similar to the status quo, with incremental adaptation and minimal yield and area changes.

**Fig. 2:**
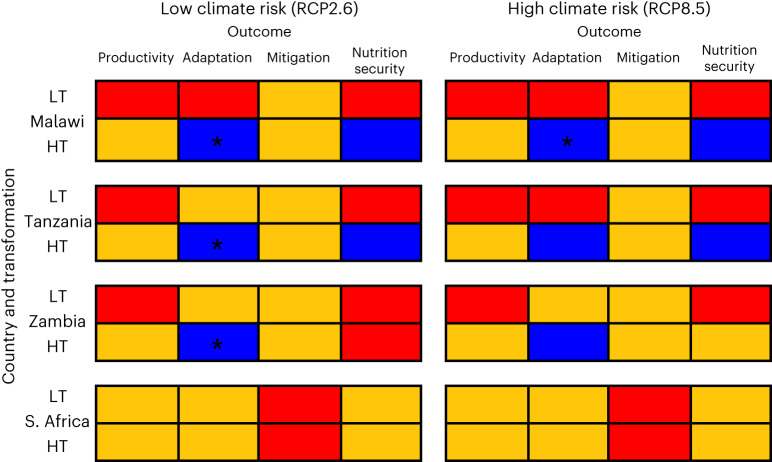
Results summary for all scenarios for the three pillars of CSA and nutrition security. LT = low-transformation scenarios (low policy efficacy in Malawi; low market connectivity in Zambia; low technological development in Tanzania; low land reform in South Africa) and HT = high transformation. RCP2.6 = low climate risk. RCP8.5 = high climate risk. The scoring system was developed to summarize iFEED results for each scenario for each aspect of climate-smart agriculture and nutrition security. SuppIementary Information provides full details of the scoring system. For each aspect of climate-smart nutrition security, blue = substantial improvement, amber = improvement inconclusive, red = clear inadequacy. Note that * indicates all aspects of productivity/adaptation/mitigation are improving/not worsening in that scenario; for nutrition security, * indicates all nutrient requirements are met for all trade vignettes.

Figure 2 summarizes climate smartness and nutrition security outcomes for each scenario. CSA outcomes are assessed by whether each aspect (productivity, adaptation and mitigation) improves or worsens relative to the baseline. Descriptive result summaries for each scenario and country are available at https://ifeed.leeds.ac.uk/ along with underlying model results and implication statements. In all four countries, agricultural transformation is a much larger driver of nutrition security and CSA outcomes than the degree of climate risk. In Malawi, Tanzania and Zambia, high population growth combines with LT conditions to reduce nutrition security. In contrast, HT scenarios show improvements to nutrition security. The South Africa high climate risk scenarios show the counter-intuitive effect of improving nutrition security. This is because stakeholder input to the scenarios indicated greater investment in adaptation under high climate risk. For example, new crop varieties, irrigation expansion and crop diversification, which lead to increased production and a more varied food supply.

In all scenarios where nutrition security improves, GHG emissions from agriculture increase due to agricultural expansion and higher yields. However, in these scenarios, soil organic carbon typically increases due to the increased organic inputs to the soil which partially compensates for the increased emissions, resulting in net emissions falling in several HT scenarios. Other analysis has shown that scenarios of intensification can result in lower emissions than scenarios of agricultural expansion^26^.

**Fig. 3:**
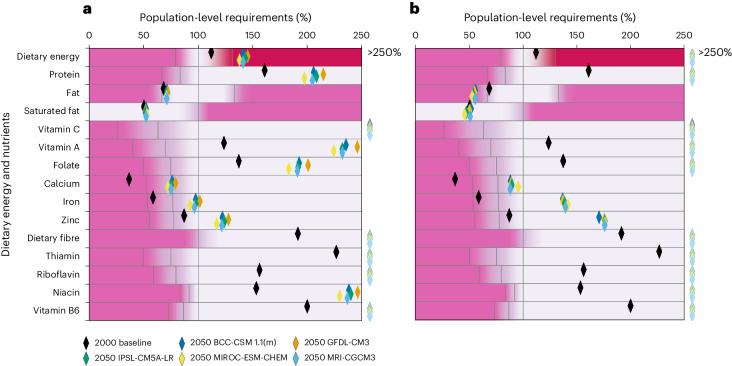
Per capita nutrient supplies with business-as-usual trade, relative to population requirements in Tanzania. **a**,**b**, Per capita nutrient supplies with business-as-usual trade, relative to population requirements (100%) for HT-RCP2.6 (**a**) and HT-RCP8.5 (**b**) in Tanzania. Black diamonds indicate baseline (2000) per capita nutrient levels. The five coloured diamonds indicate the projected outcomes in 2050 under different climate models. Grey areas indicate where per capita nutrient requirements are met, and pink areas indicate that requirements are not achieved, with intermediate areas marginal. For all nutrients other than energy and fat, the first threshold represents the Lower Reference Nutrient Intake; the second, the Estimated Average Requirement; the third, the Reference Nutrient Intake (principal target). For fat, thresholds correspond to minimum, min–max midpoint and maximum recommended intakes, respectively. For energy, the respective thresholds are minimum, average and maximum dietary energy requirements. The dark pink area indicates where calories are greater than requirements. Vitamin A is measured in retinol activity equivalents. BCC-CSM 1.1(m), Beijing Climate Center, Climate System Model, version 1.1; GFDL-CM3, Geophysical Fluid Dynamics Laboratory Climate Model, version 3; IPSL-CM5A-LR, L’Institut Pierre-Simon Laplace Coupled Model, version 5A, coupled with Nucleus for European Modelling of the Ocean (NEMO), low resolution; MIROC-ESM-CHEM, Model for Interdisciplinary Research onClimate, Earth System Model coupled with atmospheric chemistry; MRI-CGCM3, Meteorological Research Institute Coupled Atmosphere–Ocean General Circulation Model, version 3. Source data

**Fig. 4:**
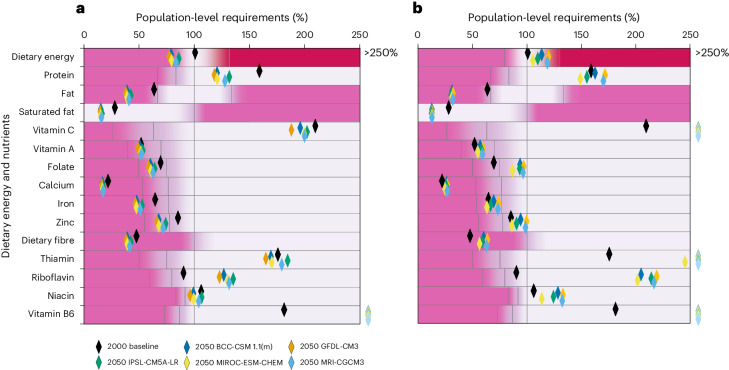
Per capita nutrient supplies with business-as-usual trade, relative to population requirements in Zambia. **a**,**b**, Per capita nutrient supplies with business-as-usual trade, relative to population requirements (100%) for HT-RCP2.6 (**a**) and HT-RCP8.5 (**b**) in Zambia. Black diamonds indicate baseline (2000) per capita nutrient levels. The five coloured diamonds indicate the projected outcomes in 2050 under different climate models. Grey areas indicate where per capita nutrient requirements are met, and pink areas indicate that requirements are not achieved, with intermediate areas marginal. For all nutrients other than energy and fat, the first threshold represents the Lower Reference Nutrient Intake; the second, the Estimated Average Requirement; the third, the Reference Nutrient Intake (principal target). For fat, thresholds correspond to minimum, min–max midpoint and maximum recommended intakes respectively. For energy, the respective thresholds are minimum, average and maximum dietary energy requirements. The dark pink area indicates where calories are greater than requirements. Vitamin A is measured in retinol activity equivalents. BCC-CSM 1.1(m), Beijing Climate Center, Climate System Model, version 1.1; GFDL-CM3, Geophysical Fluid Dynamics Laboratory Climate Model, version 3; IPSL-CM5A-LR, L’Institut Pierre-Simon Laplace Coupled Model, version 5A, coupled with Nucleus for European Modelling of the Ocean (NEMO), low resolution; MIROC-ESM-CHEM, Model for Interdisciplinary Research on Climate, Earth System Model coupled with atmospheric chemistry; MRI-CGCM3, Meteorological Research Institute Coupled Atmosphere–Ocean General Circulation Model, version 3. Source data

Figures 3 and 4 show nutrition security results for Tanzania and Zambia HT-RCP2.6 and RCP8.5 scenarios assuming business-as-usual trade; all other nutrition security results assuming business-as-usual trade are in Supplementary Text 7. In Tanzania in the baseline, bovine meat production is approximately 200,000 tonnes, and in Zambia it is approximately 50,000 tonnes. In all HT scenarios, livestock meat (including bovine meat, sheep and goat meat, pig meat and poultry meat) and dairy production (from bovine milk and sheep and goat milk) more than doubles due to a combination of increases to livestock feed from crops and livestock pasture expansion. The most common nutrients that fail to meet population requirements in these scenarios are fat, calcium and iron. This suggests that increases to livestock production—that is, increases larger than projected population increases—could help meet these requirements given low baseline livestock consumption compared with many other countries, albeit with environmental costs. This trade-off is discussed further in the ‘Policy implications’ section.

## Agricultural transformations for nutrition security

### Micronutrient-rich, productive crops for nutrition security

In all LT scenarios, incremental adaptation is insufficient to ensure an adequate nutrient supply for the population by mid-century. In the HT scenarios, transformative adaptation improves nutrition security due to increases in micronutrient-rich crops such as fruit and vegetables. Our results suggest that a continued focus on maize will continue to lead to sub-optimal nutritional outcomes in all countries.

Reduced crop diversity was considered by stakeholders to be a possible outcome in Tanzania and Zambia HT-RCP8.5 scenarios, in contrast to HT-RCP2.6 scenarios, which were associated with increased diversification. In both Tanzania and Zambia HT-RCP8.5 scenarios, the resulting focus on fewer, higher-yielding crops (such as sugar cane, onions, cassava and fruit and vegetable commodities) leads to per capita food supply exceeding requirements if assuming some degree of international trade. Whereas the increased supply of these commodities leads to increases in micronutrient-rich fruit and vegetables, there is also a substantial oversupply of calories through expansion of maize and sugar cane—for example, there are more than 250% of required per capita calories in the Tanzania HT-RCP8.5 scenario. Overproduction of calories to improve micronutrient supplies is not realistic or desirable. Supplementary analysis shows that with none of the increased sugar cane production and 50% of the maize increase seen in the 2050 HT-RCP8.5 scenario, micronutrient supplies still improve relative to the baseline due to the increase in other more nutrient-rich commodities, but even with these reductions, there was still an oversupply of calories, albeit smaller (139% of requirements; Supplementary Table 5).

With increased crop diversification in the HT-RCP2.6 scenarios, per capita nutrient supplies also improve. In Tanzania, per capita calorie and micronutrient supplies are generally inferior (iron, zinc and calcium inadequacies) compared with the HT-RCP8.5 scenario due to reduced crop production. In Zambia, increased crop diversification but lower crop production in the HT-RCP2.6 scenario results in inadequate calorie and micronutrient supply, relative to both the HT-RCP8.5 scenario and, for most nutrients, relative to the 2000 baseline, owing to population increases outpacing agricultural production.

Several studies identify a relationship between crop diversification and climate resilience^27–29^. A trade-off is evident in our results between crop diversification and crop production, with the largest increases in production associated with HT scenarios that reduce crop diversity due to expansion of the highest-yielding crops, notably maize. However, the increase in maize monocultures implied by stakeholders in scenarios of reduced crop diversity can result in greater risks from crop pests and diseases, and given reduced risk-spreading across multiple crops, fewer opportunities for on-farm income generation and greater detrimental health impacts, particularly for children, mothers and vulnerable and poor populations^29^(Supplementary Text 3). Our analysis also shows that maize is more susceptible to climate extremes than other crops, including soybean (Supplementary Text 1). Soybean is one of a number of crops important for diversification policy agendas in the region due to its important role as a cash crop and climate resilience^30^ (‘Policy implications’ section). If future food systems rely on fewer crops, there could be increased risks of obesity and associated non-communicable diseases, such as type II diabetes, cardiovascular disease and some forms of cancer^31^, continuing current trends in global food systems^32^. Thus, expansion of maize and not a diverse set of crops can have a number of negative consequences. There are challenges to expanding fruit and vegetable production, such as dealing with increased quantities of highly perishable foods, which policies need to account for^33,34^(Supplementary Text 3). There would be an increased need for infrastructural development, particularly for agricultural services such as storage, processing and transportation to cut post-harvest losses, and additionally fruit and vegetable production is commonly input and labour intensive^35^.

### Cropland expansion, yield trends and trade

Mid-century nutrient requirements remain only partially fulfilled in all scenarios, despite the transformative agricultural adaptation strategies employed in HT scenarios. To completely fulfil the populations’ nutrition requirements, further changes to domestic production or trade are necessary—for example, reconfiguring domestic food production or food import dependencies to increase the supply of specific targeted food items.

Stakeholder-designed trade vignettes explored the nutrition security consequences of altering food imports and exports in each scenario. Of the 16 scenarios across all four countries, nine of these stakeholder-designed trade vignettes have net imports (notably including all high climate risk scenarios), with more than a doubling of imports compared with business-as-usual trade in some cases. For context, in South Africa, baseline maize exports are greater than imports. Imports are approximately five times larger than the other three countries at 5 million tonnes^36^. Although import increases generally lead to higher average per capita nutrient outcomes, in most cases this is still insufficient. Therefore, unless relying on greatly increased food imports, domestic production in these countries needs to increase to fulfil calorie and micronutrient requirements. In the absence of unprecedented yield increases, the scenarios show that the supply of calories and nutrients only improve by expanding agricultural areas.

Tanzania and Malawi HT scenarios show more favourable nutrition security outcomes than the Zambia scenarios, with most nutrient requirements satisfied. In the more favourable scenarios, the factors leading to increased production are broadly the same as in Zambia HT scenarios: increases to irrigation and yields and a focus on maximizing crop production through the highest-yielding crops. The key difference is in the future expansion of agricultural land in Tanzania and Malawi: arable land expands by over 50%, and as a result, there are sufficient calories and nutrients, with the exceptions of marginally inadequate fat in Malawi and marginal fat, calcium and iron supplies in Tanzania. By contrast, Zambia crop areas expand by only 5% in HT scenarios.

It is most likely that a combination of yield improvements for more micronutrient-rich crops and area expansion will be needed to achieve nutrition security for the growing SSA population by mid-century. Yield increases in HT scenarios in Malawi, Tanzania and Zambia are on average about 150%, matching the largest increases seen in the region from 1960 to 2010. Studies suggest that greater than threefold yield gains in SSA are possible by mid-century through improved soil fertility and crop varieties^21,37^. If productivity gains are not sufficient, area is available in SSA for agricultural expansion^21,38^. There can be substantial biodiversity losses from such expansion (Supplementary Text 4), suggesting that in the future there should be prioritization of productivity gains for calories and nutrients over land-use expansion. A majority of agriculturally suitable land is already in use in Malawi^39^, more so than in Tanzania and Zambia^21,38^. Much of the non-agricultural land in Malawi consists of Miombo woodland, making expansion problematic due to loss of ecosystem services^40^. In addition, protected areas are increasingly under threat from agricultural expansion^41^. The importance of increasing productivity of micronutrient-rich crops is therefore all the more important in this context.

## Policy implications

This analysis highlights how various policy areas need to maximize synergies to improve climate-smart nutrition security in SSA by mid-century. The balance between imports and domestic production, agricultural land-use expansion and strategies to diversify and/or intensify production are key areas that require a climate-smart nutrition security lens. Whereas our findings are relevant to other SSA countries with similar climate risks and nutrition security challenges, stakeholder engagement and bespoke analyses are crucial if seeking to influence country-specific policy development.

Due to increasing food price volatility from climate^42^ and geopolitical factors^43^ such as the war in Ukraine, relying on agricultural trade for an adequate supply of calories and nutrients is an increasingly risky option. This could also be economically unrealistic, especially when there is not a diverse range of source markets to improve supply resilience^44^. The southern hemisphere is particularly at risk of crop yield instability due to climate change^45^. Consequently, if countries prioritize local production and markets—rather than rely on a globally connected food system—our analysis shows that SSA will need to increase domestic food production by mid-century given projected population growth, with a particular emphasis on commodities that will help address key nutrient deficiencies. Our results show that even with the impacts of climate change, relying on domestic food production increases—particularly of micronutrient-rich foods—with business-as-usual trade can lead to improved supplies of micronutrients. More perishable foods such as fruits and vegetables are less likely to be available from imports in any case, and therefore as these micronutrient-rich commodities are required to achieve nutrition security, it is important that domestic production strategies provide for these.

The largest differences in micronutrient supplies are across the HT vs LT scenarios, rather than across different climate change scenarios, giving further evidence that the future of nutrition security through adequate supplies of calories and nutrients is in the hands of domestic policymakers, even in the face of climate change uncertainty. That being said, the impacts of climate change extremes are important due to projected food production shocks increasing. Our results also point to sensible strategies to mitigate against these extreme impacts—for example, crop diversification as a strategy to spread risks and in particular from maize as a monocrop to reduce yield shocks, while recognizing the cultural importance of maize in the diet.

Prior studies focus mostly on production and calories, suggesting that yield gap closure is needed to maintain or increase food production for major cereal crops^20,21,46,47^. Even with yield gap closure, agricultural land expansion in SSA is needed to fulfil cereal production demand by mid-century^21^. Our analysis shows that without expansion of more diverse, micronutrient-rich crops, which provide sufficient calories and nutrients (in particular, calcium, iron, fat and zinc), achieving nutrition security is challenging in SSA even with productivity improvements. This result is supported by other studies, showing that smallholder nutrition security can be improved by diversifying away from maize despite its cultural importance^29,48,49^ and that similar nutrient deficiencies can be expected without targeted interventions^15^.

Whereas maize will continue to be an important economic and staple crop, specific policy options do exist for transitioning away from maize, such as in the Zambian policy agenda. iFEED evidence is supporting this in the development of the forthcoming Second Generation National Agriculture Investment Plan (NAIPII 2022–2026), the National Crop Diversification Strategy (2020) and the Zambia Soybean Strategy and Investment Plan (2022). The Zambian National Agricultural Policy (2004) and Second National Agricultural Policy (2016) also provide a framework for crop diversification to achieve food and nutrition security and agricultural transformation. Soybean has also recently been highlighted as a crop with expansion potential across Africa^30^, primarily as a cash crop and a source of livestock feed. Our findings suggest that investment in soybean as an emergent crop has potential benefits for improving climate resilience and nutrition security through both direct consumption and as a source of livestock feed, increasing the supply of animal-based foods. Additionally, cash crops can have benefits for nutrition security that staple crops do not provide, such as increasing income and therefore access to a more diverse range of foods^50^. Alongside increases in productivity of micronutrient-rich crops, cash crops will continue to form a crucial part of incomes, and without adequate planning, communities can adopt unsustainable alternative practises such as encroachment on protected areas through pastoral expansion^51^.

There is a need to rebalance livestock consumption globally given overconsumption in many high-income countries^52^ and the lack of key micronutrients in SSA diets^49^. Whereas livestock production is associated with increased emissions and places high demands on land and water (Supplementary Text 4), it also provides essential micronutrients that are currently deficient in many people in SSA^52^. Historically, agricultural land-use change has been driven by both population growth and increasing demands for animal products^53^. Increased production and consumption of animal-based products in the region could reduce nutrient gaps but should not aim to reach the unsustainable production levels currently seen in the Global North. Following the trends in dietary changes with nutrition transition through economic development, seen in many low- and middle-income countries, it is likely that consumption of animal products will increase^32^. Given the relatively low GHG emissions in SSA^54^, and the challenges associated with achieving nutrition security by mid-century, policies should focus on providing sufficient food to meet nutrient requirements if faced with the trade-off between increasing emissions and avoiding food and nutrition insecurity, and arguably some increases in emissions could be regarded as tolerable. In any case, without domestic food production increases, emissions would be outsourced if relying on increased imports. Crop breeding for biofortification^55^ and increased production of crops such as millet and sorghum can contribute to alleviating calcium, iron and zinc shortfalls^56,57^ and reduce demands on land and water. Expansion of such traditional and neglected crops will require increased scientific and market investment, however.

Optimizing climate resilience and nutrient supplies requires that crop-specific investments are not pursued in isolation but are grounded in holistic food system strategies. At country scales and above, studies that explore food system transformation are limited to providing assessments on future food^21^ or nutrition^15^ security. None of these analyses quantify impacts on climate smartness, despite this being a key component of complex trade-offs inherent to food system transformation^58^. Here we provide a comprehensive assessment of the transitions needed for climate-smart nutrition security.

There are opportunities to focus on commodities that are more climate resilient and nutritionally important, and if climate-smart practises can increase productivity while minimizing environmental impacts, policies can be designed to benefit social, environmental and nutrition security objectives. Additional agricultural inputs and access to improved seed varieties are necessary for yield gap closure in SSA^37^; addressing crop nutrient deficiencies alone could lead to 50% of yield gap closure^59^. Climate finance can help with the costs of such a transition, although more needs to be done to ensure that funds address productivity gains and climate change impacts on the most vulnerable^60^. For example, farmer insurance schemes could help to deal with increasing climate variability and boost productivity^61^. Crucially, the social, health and environmental benefits of transitioning to new diets are projected to be substantial^62^, highlighting the need to consider the benefits of transitions to more nutrient-secure diets to incentivize the public and private sectors to fund necessary transformations.

Without holistic approaches, adaptation will continue to be sub-optimal from health and environmental perspectives^18,19^. The greater the focus on sustainable productivity increases that target nutrient requirements, the smaller the requirements for agricultural area expansion, increased emissions and damaging environmental impacts^63–65^.

## Methods

Note that iFEED methods and limitations have previously been fully described^22^, so a concise summary of the steps towards climate-smart nutrition security scenario assessment is provided here. We also provide further comparison with other integrated modelling approaches in Supplementary Text 5. Our modelling does not account for increased costs of production, instead focusing on the benefits of various adaptation decisions. This is because we do not advocate implementing any specific scenario but instead seek to compare the positives and negatives of various scenarios to point towards robust pathways of change, which culminate in climate-smart nutrition security. Through post hoc discussion with stakeholders, these results can be used to inform agricultural policy development that is cognizant of the costs involved in seeking to implement desirable transformations.

### Stakeholder-defined scenarios

First, a scenario exercise is used to explore the range of possibilities that the future may hold^11,12^. Our analysis compares a baseline centred on 2000 (1990 to 2010) with a future centred on 2050 (2040 to 2060). Food system stakeholders identify a set of driving forces that shape future food system outcomes. Through discussion, two independent and impactful drivers (described as critical uncertainties) are selected for which there is high uncertainty, thus maximizing the range of possible futures explored. The two critical uncertainties are used to create a 2 × 2 matrix that frames four potential future scenarios. Figure 1 summarizes the scenarios for each country. In all countries, the level of climate risk was selected as one critical uncertainty, with low-climate-risk scenarios being characterized by RCP2.6 and high-climate-risk scenarios characterized by RCP8.5. In Malawi, South Africa, Tanzania and Zambia, respectively, the other critical uncertainty selected was the effectiveness of policy implementation (the degree to which agricultural and food system policies will be systemic, aligned, well-implemented and adopted, enabling progressive, nutritionally adequate and sustainable food system outcomes), the extent of land reform (from minor adjustments compared with today, to extensive ‘land restitution’ to empower farm workers and reduce inequality), the extent of technological transformation (the degree to which general improvements in productivity from better implementation of agricultural technologies have taken place) and the degree of market connectivity and functionality (how connected international and domestic food system markets are to Zambia’s agricultural system; technology was also an important factor linked with market connectivity). The scenarios with a high degree of change in this second critical uncertainty are known as ‘high-transformation’ (HT) scenarios and the opposing scenarios known as ‘low-transformation’ (LT) scenarios. Full details of the stakeholder scenario workshops are at https://africap.info/reports/.

Stakeholders inform the modelling of these scenarios in terms of changes to crop yields, agricultural areas, crop varieties and diversity, irrigation and international trade. We represented increased/decreased crop diversification as a decreased/ increased fraction of total cropped area taken by maize and more/fewer crops sharing the majority of cropped areas. HT scenarios generally assumed a continuation of historical yield trends in the region, representing an optimistic view of future crop yields based on observed data. Crops were spatially distributed within each country to maximize production in these scenarios—that is, optimization to maximize crop production given the prescribed crop area and yields.

Dietary demand trends in lower- and middle-income countries are towards increasing consumption of ultra-processed foods and meat products. It is uncertain to what extent demand will shift in SSA towards ‘westernization’ of diets by 2050, although current trends are towards increased consumption of ultra-processed foods and meat and dairy^32^.

Whereas our modelling framework does not explicitly account for changes in demand, such trends in diets drive changes in food production systems. All high-transformation scenarios include increased livestock production, primarily to explore how nutrition security could be ensured by mid-century but also reflecting stakeholder recognition of known trends towards increased demand for livestock products, which informed the projections of future land use. In addition, trade vignettes cover a full range of trade possibilities, from self-sufficiency to stakeholder assessment of future imports and exports in each scenario, thus implicitly including any expected changes in demand. Therefore, whereas the focus of the analysis is explicitly on how agricultural transformation (via domestic policy decisions) could help deliver nutrition security (and what the implications of these transitions would be for climate smartness), changes in demand inherently underpin stakeholder assumptions around future production and trade.

### Integrated modelling of climate, food and emissions

Integrated modelling provides each scenario with quantification of changes to crop and livestock production. All crop commodities grown in each country in the baseline (1990 to 2010) are included in the food production and nutrition security analysis. Crop production changes are calculated from yield and area changes specific to each scenario. Crop yield changes are the result of simulated climate change impacts using the General Large Area Model for annual crops^66^ and yield trends applied as agreed with stakeholders. For each crop, continuation of historical trends as seen in Food and Agriculture Organization of the United Nations Statistics Division (FAOSTAT) yield data^36^ from 1960 to 2010 were applied in Malawi, Tanzania and Zambia HT scenarios. LT scenarios in these three countries assume no yield trend applied and only autonomous adaptation to climate change (consisting of changes to planting dates and crop varieties, although only those varieties that are currently available). All South Africa scenarios assumed an intermediate yield trend for each crop, being half of the historical trend. HT scenarios in all four countries accounted for adaptation to climate change in the form of changing of planting dates and new crop varieties that account for any warming-induced reduction in the length of the growing season. Area changes are also scenario specific and determined in conjunction with stakeholders (Fig. 1); maximum possible increases were determined using Land-Use Harmonization II data^67^ and assumed all land was available for agricultural expansion if not forested, urban or protected according to The World Database on Protected Areas^68^.

Livestock production changes are calculated using projected changes to livestock pasture, crop residues and crop production used as livestock feed and assuming historical relationships between livestock feed and livestock meat and dairy production remain the same by 2050. These relationships are calculated using data^69^ in the following categories: bovine meat, bovine milk, sheep and goat meat, sheep and goat milk, pig meat, poultry meat and eggs.

Nutrition security (defined here as adequate energy and nutrient supplies to meet dietary requirements at a population level, noting that we do not assess the distribution or access of food within the population) was quantified for each scenario given domestic food production changes, assuming medium-variant United Nations population projections for 2050 and contrasting trade scenarios referred to as trade vignettes: self-sufficiency (assuming no imports or exports and thus addressing how well domestic production matches domestic requirements); business as usual (imports and exports remaining in the same proportions to domestic production as at baseline) and stakeholder expectations (reflecting in-country expert judgements about likely future trade dependencies).

The FAOSTAT Food Balance Sheet (FBS) data provide an estimate of the supply of 96 food commodities based on domestic production, imports and exports, including stock variation of each commodity within each country. These data are further categorized into supply for human consumption and other uses (for example, feed, seed and losses). Although they provide an estimate of per capita supply of calories, protein and fat, data for micronutrients are not supplied in the FBS, therefore in iFEED the supply of energy and all nutrients are calculated for each country using an internally consistent method^70^. FBS food commodities are converted to food as eaten, adjusting for unavoidable waste (for example, inedible peel, bones) and household waste (for example, edible food). The food commodities are disaggregated into food items and matched to foods in country or region-specific food composition tables, which provide an estimate of the supply of calories, protein, fat, carbohydrate, saturated fat, fibre, calcium, zinc, iron, vitamin C, thiamin, riboflavin, niacin, folate and vitamin B6. Each food item is then weighted to represent the quantity of each food eaten at a country level, before being aggregated back to food commodity groups. We assume no changes to the weightings of foods within each food item between baseline and future for this calculation. Although dietary composition is likely to change, many of the changes may be expected to be between rather than within food items, although the rate and extent of this transition is uncertain. More generally, whereas changes to diets in these countries are likely with economic development to move through a nutrition transition to those observed in high-income countries^32^, our focus was on food supply rather than demand so we have not commented on potential dietary changes for the weighting calculation. Lastly, total nutrient supplies are calculated. The marker of adequate nutrition supply is set to achieving the supply of population-level nutrient requirements taken from World Health Organization recommendations. The population-level nutrient requirements are country specific and adjusted for projected demographic changes (population size, age, sex and fertility rates) based on medium-variant United Nations projections to 2050.

We quantified changes to greenhouse gas emissions, soil organic carbon and climate extremes to holistically assess climate-smart nutrition security. Extremes of climate change are analysed in terms of changes to extremes of temperature and precipitation and resulting impacts on crop yield shocks (that is, years with approximately half of the mean baseline yield). Model results are summarized using calibrated statements—concise summaries that are associated with an assessment of confidence in model outcomes based on comparisons to the literature and expert judgement of model result uncertainty.

### Integration of expert judgement and result summary process

Critical analysis of model outputs is undertaken by social, ecological and environmental scientists, who use the calibrated statements as the basis for implication statements. This allows iFEED to explore broader food system implications than models can alone; for example, how changes to agricultural land use and crop diversification might impact pest and disease risks, soil health, inequality and land-use conflict. The calibrated and implication statements are collated at the level of each scenario and then for each country, providing descriptive scenario and country-level summaries. These are available to view at https://ifeed.leeds.ac.uk/.

A scoring system was developed to summarize iFEED results for each scenario for each aspect of climate-smart agriculture and nutrition security, the results of which are shown in Fig. 2. Supplementary Text 6 provides full details of the scoring system. For each aspect of climate-smart nutrition security:

- Blue = substantial improvement
- Amber = improvement inconclusive (either not a substantial change or trade-offs to improvements possible)
- Red = clear inadequacy

Following this assessment of each scenario, cross-scenario comparisons are made to draw out the commonalities that lead to improvements in nutrition security and climate smartness. Using these cross-scenario comparisons, policy implications are co-developed with stakeholders by incorporating country-specific policy context with the integrated assessment outputs.

### Reporting summary

Further information on research design is available in the Nature Portfolio Reporting Summary linked to this article.

### Supplementary information

Supplementary Information Supplementary Text, Tables 1–6 and Figs. 1–11.

Reporting Summary

### Source data

Source Data Fig. 3 Data associated with Fig. 3 (Tanzania nutrition security HT scenarios).

Source Data Fig. 4 Data associated with Fig. 4 (Zambia nutrition security HT scenarios).

## Data Availability

Source data supporting conclusions are shown in Supplementary Tables 1-4. Input data used in this study are from publicly available sources and referenced in Jennings et al. (2022). In summary, these consist of: the cumulative distribution function transform bias-corrected CMIP5 data over Africa at http://amma2050.ipsl.upmc.fr/ (to access the data, users must contact the lead author at moflod@locean-ipsl.upmc.fr); FAOSTAT yield and area and Food Balance Sheet data from https://www.fao.org/faostat/; soil data from the Regridded Harmonized World Soil Database v 1.2 at https://daac.ornl.gov/SOILS/guides/HWSD.html and gridded area data from Land-Use Harmonization II and World Database on Protected Areas. Source data are provided with this paper.

## Code availability

The methods used have been previously fully described in Jennings et al. (2022). The General Large Area Model for annual crops was used for the crop yield simulations. An older version of this model is available online at https://licensing.leeds.ac.uk/product/general-large-area-model-for-annual-crops-glam. The version (version number 79e1615) used for the simulations in this paper is available upon reasonable request. The ECOSSE model (Estimating Carbon in Organic Soils-Sequestration and Emissions) provided projections of greenhouse gas emissions, soil organic carbon and nitrogen dynamics associated with agriculture in each future scenario, taking into account yield and land-use changes. A spatial version of ECOSSE-Global ECOSSE (version 6.2b) was used. More information is available at https://soil-modeling.org/resources-links/model-portal/ecosse. An Excel spreadsheet was developed for nutrition data analysis and is available upon reasonable request.

## References

[1] Vermeulen SJ (2013). Addressing uncertainty in adaptation planning for agriculture. Proc. Natl Acad. Sci. USA.

[2] IPCC *Special Report on Climate Change, Desertification, Land Degradation, Sustainable Land Management, Food Security, and Greenhouse Gas Fluxes in Terrestrial Ecosystems (SR2): Background Report for the Scoping Meeting* (WMO, 2017).

[3] Horton P (2017). An agenda for integrated system-wide interdisciplinary agri-food research. Food Secur..

[4] Challinor AJ, Arenas-Calles LN, Whitfield S (2022). Measuring the effectiveness of climate-smart practices in the context of food systems: progress and challenges. Front. Sustainable Food Syst..

[5] Hellin J (2022). Transformative adaptation and implications for transdisciplinary climate change research. Environ. Res. Clim..

[6] Fedele G, Donatti CI, Harvey CA, Hannah L, Hole DG (2019). Transformative adaptation to climate change for sustainable social-ecological systems. Environ. Sci. Policy.

[7] Springmann M (2017). Mitigation potential and global health impacts from emissions pricing of food commodities. Nat. Clim. Change.

[8] Stehfest E (2019). Key determinants of global land-use projections. Nat. Commun..

[9] Hasegawa T (2018). Risk of increased food insecurity under stringent global climate change mitigation policy. Nat. Clim. Change.

[10] Nelson GC (2014). Climate change effects on agriculture: economic responses to biophysical shocks. Proc. Natl Acad. Sci. USA.

[11] O’Neill BC (2020). Achievements and needs for the climate change scenario framework. Nat. Clim. Change.

[12] Benton TG (2019). Using scenario analyses to address the future of food. EFSA J..

[13] Riahi K (2017). The shared socioeconomic pathways and their energy, land use, and greenhouse gas emissions implications: an overview. Glob. Environ. Change.

[14] Wiebe K (2015). Climate change impacts on agriculture in 2050 under a range of plausible socioeconomic and emissions scenarios. Environ. Res. Lett..

[15] Nelson G (2018). Income growth and climate change effects on global nutrition security to mid-century. Nat. Sustain..

[16] Arenas-Calle LN, Whitfield S, Challinor AJ (2019). A Climate Smartness Index (CSI) based on greenhouse gas intensity and water productivity: application to irrigated rice. Front. Sustain. Food Syst..

[17] Tripathi HG (2022). Climate-smart agriculture and trade-offs with biodiversity and crop yield. Front. Sustainable Food Syst..

[18] de Ruiter H, Macdiarmid JI, Matthews RB, Smith P (2018). Moving beyond calories and protein: micronutrient assessment of UK diets and land use. Glob. Environ. Change.

[19] Ambikapathi R (2022). Global food systems transitions have enabled affordable diets but had less favourable outcomes for nutrition, environmental health, inclusion and equity. Nat. Food.

[20] Schneider JM, Zabel F, Schünemann F, Delzeit R, Mauser W (2022). Global cropland could be almost halved: assessment of land saving potentials under different strategies and implications for agricultural markets. PLoS ONE.

[21] Van Ittersum MK (2016). Can sub-Saharan Africa feed itself?. Proc. Natl Acad. Sci. USA.

[22] Jennings, S.A. et al. A new integrated assessment framework for climate-smart nutrition security in sub-Saharan Africa: the integrated Future Estimator for Emissions and Diets (iFEED). *Front. Sustainable Food Syst.*10.3389/fsufs.2022.868189 (2022).

[23] Webber H, Gaiser T, Ewert F (2014). What role can crop models play in supporting climate change adaptation decisions to enhance food security in sub-Saharan africa?. Agric. Syst..

[24] Hamilton SH, ElSawah S, Guillaume JH, Jakeman AJ, Pierce SA (2015). Integrated assessment and modelling: overview and synthesis of salient dimensions. Environ. Modell. Software.

[25] Famien AM (2018). A bias-corrected CMIP5 dataset for Africa using the CDF-t method: a contribution to agricultural impact studies. Earth Syst. Dyn..

[26] van Loon MP (2019). Impacts of intensifying or expanding cereal cropping in sub-Saharan Africa on greenhouse gas emissions and food security. Glob. Change Biol..

[27] Clay N, Zimmerer KS (2020). Who is resilient in Africa’s green revolution? Sustainable intensification and climate smart agriculture in Rwanda. Land Use Policy.

[28] Abberton M (2016). Global agricultural intensification during climate change: a role for genomics. Plant Biotechnol. J..

[29] Mango N, Makate C, Mapemba L, Sopo M (2018). The role of crop diversification in improving household food security in central Malawi. Agric. Food Secur..

[30] Foyer CH (2019). Modelling predicts that soybean is poised to dominate crop production across africa. Plant Cell Environ..

[31] Haslam DW, James WPT (2005). Obesity. Lancet.

[32] Popkin BM, Adair LS, Ng SW (2012). Global nutrition transition and the pandemic of obesity in developing countries. Nutr. Rev..

[33] Mason-D’Croz D (2019). Gaps between fruit and vegetable production, demand, and recommended consumption at global and national levels: an integrated modelling study. Lancet Planet. Health.

[34] Siegel KR, Ali MK, Srinivasiah A, Nugent RA, Narayan KV (2014). Do we produce enough fruits and vegetables to meet global health need?. PLoS ONE.

[35] Sachdeva S, Sachdev TR, Sachdeva R (2013). Increasing fruit and vegetable consumption: challenges and opportunities. Indian J. Community Med..

[36] *FAOSTAT: FAO Statistical Databases* (FAO, 2020); https://www.fao.org/faostat/en/#data

[37] Sanchez PA (2015). En route to plentiful food production in Africa. Nat. Plants.

[38] Chamberlin J, Jayne T, Headey D (2014). Scarcity amidst abundance? Reassessing the potential for cropland expansion in Africa. Food Policy.

[39] Li G, Messina JP, Peter BG, Snapp SS (2017). Mapping land suitability for agriculture in Malawi. Land Degrad. Dev..

[40] Ryan CM (2016). Ecosystem services from southern African woodlands and their future under global change. Philos. Trans. R. Soc. B.

[41] Meng Z (2023). Post-2020 biodiversity framework challenged by cropland expansion in protected areas. Nat. Sustain..

[42] Iizumi T (2014). Impacts of El Niño Southern Oscillation on the global yields of major crops. Nat. Commun..

[43] Anderson K (2012). Government trade restrictions and international price volatility. Glob. Food Secur..

[44] Zorya, S. et al. in *Trade Policy and Food Security* (eds Gillson, I. & Fouad, A.) 65–85 (World Bank, 2014).

[45] Iizumi T (2014). Historical changes in global yields: major cereal and legume crops from 1982 to 2006. Glob. Ecol. Biogeogr..

[46] Zhu P (2022). Warming reduces global agricultural production by decreasing cropping frequency and yields. Nat. Clim. Change.

[47] Ten Berge HF (2019). Maize crop nutrient input requirements for food security in sub-Saharan Africa. Glob. Food Secur..

[48] Lovo S, Veronesi M (2019). Crop diversification and child health: empirical evidence from Tanzania. Ecol. Econ..

[49] Galani Y, Orfila C, Gong Y (2022). A review of micronutrient deficiencies and analysis of maize contribution to nutrient requirements of women and children in Eastern and Southern Africa. Crit. Rev. Food Sci. Nutr..

[50] Longhurst R (1988). Cash crops, household food security and nutrition. IDS Bull..

[51] Baudron F, Guerrini L, Chimimba E, Chimusimbe E, Giller KE (2022). Commodity crops in biodiversity-rich production landscapes: friends or foes? The example of cotton in the Mid Zambezi Valley, Zimbabwe. Biol. Conserv..

[52] Mehrabi Z, Gill M, Wijk MV, Herrero M, Ramankutty N (2020). Livestock policy for sustainable development. Nat. Food.

[53] Alexander P (2015). Drivers for global agricultural land use change: the nexus of diet, population, yield and bioenergy. Glob. Environ. Change.

[54] Crippa M (2021). Food systems are responsible for a third of global anthropogenic GHG emissions. Nat. Food.

[55] Msungu SD, Mushongi AA, Venkataramana PB, Mbega ER (2022). A review on the trends of maize biofortification in alleviating hidden hunger in sub-Sahara Africa. Sci. Hortic..

[56] Mahendrakar MD (2019). Genetic variability, genotype × environment interaction and correlation analysis for grain iron and zinc contents in recombinant inbred line population of pearl millet [*Pennisetum glaucum* (L). R. Br.]. Indian J. Genet. Plant Breed..

[57] Devi PB, Vijayabharathi R, Sathyabama S, Malleshi NG, Priyadarisini VB (2014). Health benefits of finger millet (*Eleusine coracana* L.) polyphenols and dietary fiber: a review. J. Food Sci. Technol..

[58] Cammarano D (2023). Models can enhance science–policy–society alignments for climate change mitigation. Nat. Food.

[59] Mueller ND (2012). Closing yield gaps through nutrient and water management. Nature.

[60] Roberts JT (2021). Rebooting a failed promise of climate finance. Nat. Clim. Change.

[61] Gatti N (2023). Is closing the agricultural yield gap a “risky" endeavor?. Agric. Syst..

[62] Chichaibelu BB, Bekchanov M, von Braun J, Torero M (2021). The global cost of reaching a world without hunger: Investment costs and policy action opportunities. Food Policy.

[63] Williams DR (2021). Proactive conservation to prevent habitat losses to agricultural expansion. Nat. Sustain..

[64] van Soesbergen A (2017). Exploring future agricultural development and biodiversity in Uganda, Rwanda and Burundi: a spatially explicit scenario-based assessment. Reg. Environ. Change.

[65] Palazzo A (2017). Linking regional stakeholder scenarios and shared socioeconomic pathways: quantified West African food and climate futures in a global context. Glob. Environ. Change.

[66] Challinor AJ, Wheeler TR, Craufurd PQ, Slingo JM, Grimes DIF (2004). Design and optimisation of a large-area process-based model for annual crops. Agric. For. Meteorol..

[67] Hurtt GC (2011). Harmonization of land-use scenarios for the period 1500–2100: 600 years of global gridded annual land-use transitions, wood harvest, and resulting secondary lands. Climatic Change.

[68] *Protected Planet: The World Database on Protected Areas (WDPA)* (UNEP-WCMC & IUCN, 2019); www.protectedplanet.net

[69] Herrero M (2013). Biomass use, production, feed efficiencies, and greenhouse gas emissions from global livestock systems. Proc. Natl Acad. Sci. USA.

[70] Macdiarmid JI, Clark H, Whybrow S, De Ruiter H, McNeill G (2018). Assessing national nutrition security: the UK reliance on imports to meet population energy and nutrient recommendations. PLoS ONE.

