# Author Correction: Stakeholder-driven transformative adaptation is needed for climate-smart nutrition security in sub-Saharan Africa

**DOI:** 10.1038/s43016-024-00988-x

**Published:** 2024-04-29

**Authors:** Stewart Jennings, Andrew Challinor, Pete Smith, Jennie I. Macdiarmid, Edward Pope, Sarah Chapman, Catherine Bradshaw, Heather Clark, Sylvia Vetter, Nuala Fitton, Richard King, Sithembile Mwamakamba, Tshilidzi Madzivhandila, Ian Mashingaidze, Christian Chomba, Masiye Nawiko, Bonani Nyhodo, Ndumiso Mazibuko, Precious Yeki, Pamela Kuwali, Alfred Kambwiri, Vivian Kazi, Agatha Kiama, Abel Songole, Helen Coskeran, Claire Quinn, Susannah Sallu, Andrew Dougill, Stephen Whitfield, Bill Kunin, Nalishebo Meebelo, Andrew Jamali, Dhaquirs Kantande, Prosper Makundi, Winfred Mbungu, Frank Kayula, Sue Walker, Sibongile Zimba, Joseph Hubert Galani Yamdeu, Ndashe Kapulu, Marcelo Valadares Galdos, Samuel Eze, Hemant Tripathi, Steven Sait, Stefan Kepinski, Emmanuel Likoya, Henry Greathead, Harriet Elizabeth Smith, Marcelin Tonye Mahop, Helen Harwatt, Maliha Muzammil, Graham Horgan, Tim Benton

**Affiliations:** 1 Institute for Climate and Atmospheric Science, School of Earth and Environment, University of Leeds, Leeds, United Kingdom; 2 Institute of Biological and Environmental Sciences, University of Aberdeen, Aberdeen, United Kingdom; 3 The Rowett Institute, School of Medicine, Medical Sciences and Nutrition, University of Aberdeen, Aberdeen, United Kingdom; 4 Hadley Centre, Met Office, Exeter, United Kingdom; 5 The Global Systems Institute, University of Exeter, Exeter, United Kingdom; 6 Institute of Applied Health Sciences, School of Medicine, Medical Sciences and Nutrition, University of Aberdeen, Aberdeen, United Kingdom; 7 Chatham House, The Royal Institute of International Affairs, London, United Kingdom; 8 Food, Agriculture and Natural Resources Policy Analysis Network, Pretoria, South Africa; 9 Agricultural Consultative Forum, Lusaka, Zambia; 10 National Agricultural Marketing Council, Pretoria, South Africa; 11 Civil Society Agriculture Network, Lilongwe, Malawi; 12 Economic and Social Research Foundation, Dar es Salaam, Tanzania; 13 School of Biology, Faculty of Biological Sciences, University of Leeds, Leeds, United Kingdom; 14 Sustainability Research Institute, School of Earth and Environment, University of Leeds, Leeds, United Kingdom; 15 Regional Network of Agricultural Policy Research Institutes, Lusaka, Zambia; 16 Malawi National Planning Commission, Lilongwe, Malawi; 17 Concern Worldwide, Lilongwe, Malawi; 18 Environmental Management Unit, Ministry of Agriculture, Dodoma, Tanzania; 19 Sokoine University of Agriculture, Morogoro, Tanzania; 20 Kaypro Research Institute, Lusaka, Zambia; 21 Agricultural Research Council, Pretoria, South Africa; 22 University of the Free State, Bloemfontein, South Africa; 23 Lilongwe University of Agriculture and Natural Resources, Lilongwe, Malawi; 24 School of Food Science and Nutrition, University of Leeds, Leeds, United Kingdom; 25 Section of Natural and Applied Sciences, School of Psychology and Life Sciences, Canterbury Christ Church University, Canterbury, United Kingdom; 26 Sustainable Soils and Crops, Rothamsted Research, Harpenden, United Kingdom; 27 Department of Agriculture and Environment, Harper Adams University, Newport, United Kingdom; 28 UN Environment Programme, World Conservation Monitoring Centre (UNEP-WCMC), Cambridge, United Kingdom; 29 USAID West Africa Biodiversity and Low Emissions Development (WABiLED) Programme, Accra, Ghana; 30 Biomathematics and Statistics Scotland, Aberdeen, United Kingdom

**Keywords:** Climate-change impacts, Climate-change mitigation, Climate-change policy, Developing world, Agriculture

Correction to: *Nature Food* 10.1038/s43016-023-00901-y, published online 2 January 2024.

In the version of the article initially published, the XDER threshold for dietary energy in Figs. 3 and 4 and Supplementary Figs. 6–11 was incorrectly set at approximately 230%. This is now corrected to be at approximately 130%. Figs. 3 and 4 have been corrected in the HTML and PDF versions of the article, and amended Supplementary Information is available online.

