# Supplementary material for: Stakeholder-driven transformative adaptation is needed for climate-smart nutrition security in sub-Saharan Africa
Source: Nat Food. 2024 Jan 2;5(1):37–47. doi: 10.1038/s43016-023-00901-y (PMC10810754; doi:10.1038/s43016-023-00901-y)
Supplement: Supplementary file 1 — Supplementary Text, Tables 1–6 and Figs. 1–11. [file 43016_2023_901_MOESM1_ESM.pdf]

nature food

Article

https://doi.org/10.1038/s43016-023-00901-y

Stakeholder-driven transformative adaptation is needed for climate-smart nutrition security in sub-Saharan Africa

In the format provided by the authors and unedited

1 Climate extremes and yield shocks

To support adaptation strategies, climate model simulations assessed the likelihood of experiencing climate extremes for the present day and both low and high climate risk 2050 scenarios. Record-breaking hot and dry extremes are already possible in the present day [1], and have increased since at least the 1980s. If they occurred, these events would result in heat stress and drought conditions that could have severe impacts on agricultural systems and the people who depend on them.

For the 2050s, climate model projections across both low and high risk climate scenarios indicate a continuing increase in the frequency and severity of climate extremes, including the number of days over 35°C. Although rainfall trends are generally less clear, climate models show a trend towards more frequent and severe droughts, droughts combined with high temperatures, and more intense rainfall, which would result in increased flood risk and soil erosion. These increases are projected to be larger with RCP8.5 (see Figures 2 to 5).

Yield shocks typically increase unless assuming historical yield trends will continue to 2050 - for example, more than a doubling of the number of maize yield shocks with RCP8.5 in Malawi and Tanzania (see below calibrated statements). This result is in keeping with other recent studies on climate change impacts on yield shocks showing increasing likelihood of shocks, in particular in high emission scenarios [2].

Modeling experts summarised key results using calibrated statements (CS). Each CS is associated with a confidence assessment. Confidence is expressed in terms of the robustness of the evidence (based on any available quantifications of uncertainty, such as across climate model inputs, and the expert judgement of the modelers) and agreement of results with other work (based on literature assessment). An aggregate assessment of confidence is then made for each CS, combining both robustness and agreement assessments. A full description of calibrated statement methods has been previously published [3]. All statements are freely available online (https://ifeed.leeds.ac.uk/).

<u>Robustness assessment:</u> Medium robustness is selected for all scenarios and countries. Between 0 and 3 climate model projections are outliers in all cases, which means medium or highly robust with respect to climate model uncertainty in all cases; however, there is some uncertainty associated with crop model parameterisation also (e.g CO2 and response to duration, which differ across

crop models; and only one crop model was run here). Therefore, the robustness assessment has been downgraded to medium.

Literature summary and agreement assessment: Of the studies that report changes to crop yield variability or crop yield shock / failure rates, most project increases, although there are some exceptions, usually arising due to uncertainty in rainfall projections (e.g. see 4). Most typically crop variability (and therefore, yield shocks / failures) are projected to increase but some studies suggest that decreases in variability are possible (see for example the 5 meta-analysis). Therefore, if all projections in iFEED shows an increase in baseline threshold crop yield shocks in terms of number of years, high agreement is selected. If the iFEED ranges overlap with 0 / show a decrease in crop yield shock rate in some cases, medium agreement is selected. If all projections indicate a decrease in crop yield shock, low agreement is selected.

Malawi LT-RCP8.5 maize: Crop yield shocks increase by 174.5% on average (range 63.2 to 368.2%), from approximately 1.6/21 years in the baseline to 5.2/21 years in the future. This is caused by decreasing mean yields, bringing the yields closer, on average, to the threshold for crop yield shock.

High confidence (medium robustness and high agreement)

Malawi LT-RCP8.5 soybean: Crop yield shocks increase by 28.5% on average (range -80 to 261%), from approximately 1.1/21 years in the baseline to 1.6/21 years in the future. This is caused by decreasing mean yields, bringing the yields closer, on average, to the threshold for crop yield shock. Increasing yield variability also results in increased crop yield shock.

Medium confidence (medium robustness and medium agreement)

Tanzania LT-RCP8.5 maize: Crop yield shocks increase by 128% on average (range 17.1 to 356%), from approximately 1.7/21 years in the baseline to 4/21 years in the future. This is caused by decreasing mean yields, bringing the yields closer, on average, to the threshold for crop yield shock.

High confidence (medium robustness and high agreement)

Tanzania LT-RCP8.5 soybean:

Crop yield shocks increase by 28.1% on average (range -43.8 to 97.1%), from approximately 1.2/21 years in the baseline to 1.5/21 years in the future. This is caused by decreasing mean yields, bringing the yields closer, on average,

to the threshold for crop yield shock. Increasing yield variability also results in increased crop yield shock.

Medium confidence (medium robustness and medium agreement)

2 Climate impacts - average changes in yields to 2050

In Malawi, Tanzania, and Zambia, LT scenarios are associated with worsening nutrition security (e.g. see Figure Supplementary Information 6A for the Malawi LT-RCP2.6 scenario) and feature only incremental adaptation rather than more transformative changes (altering what crops are grown, and where). Whilst projections show that incremental adaptation can benefit yields by as much as 30% with high climate risk in South Africa compared to projections that assume no changes to planting dates and varieties by 2050 - without more transformative adaptation strategies, nutrition security outcomes worsen by 2050 given the projected three-fold population growth in these countries.

Crop model simulations varied in terms of adaptation to climate change through changes to crop varieties, irrigation and planting dates. Incremental adaptation was simulated by allowing planting dates and crop varieties to change (restricted to those varieties that were available during the baseline period, with varieties defined using different thermal time phenological parameters, i.e. different maturity classes).

Modeling experts summarised key results using calibrated statements (CS). Each CS is associated with a confidence assessment. Confidence is expressed in terms of the robustness of the evidence (based on any available quantifications of uncertainty, such as across climate model inputs, and the expert judgement of the modelers) and agreement of results with other work (based on literature assessment). An aggregate assessment of confidence is then made for each CS, combining both robustness and agreement assessments. A full description of calibrated statement methods has been previously published [3]. All statements are freely available online (https://ifeed.leeds.ac.uk/).

South Africa LT-RCP8.5 maize without adaptation:

The mean percentage change to maize yield with no adaptation for RCP8.5 is -43% (range across climate models -59 to -31%; 0/18 climate models are outliers).

Medium confidence (medium robustness and medium agreement).

Robustness assessment: Medium robustness. 0/18 climate models are outliers, which means highly robust with respect to climate model uncertainty; however, there is some uncertainty associated with crop model parameterisation (e.g CO2 and response to duration, which differ across crop models; and only one crop model was run here). Therefore, the robustness assessment has been downgraded to medium.

Agreement assessment: Many studies in the literature suggest that maize yields are likely to decline with climate change, and this decline is greater without any form of adaptation. However, the lower end of the range (ie -59%) is nearly an outlier compared to previously published meta-analysis [5]. This suggests the wording "losses of upwards of 30%", rather than the citing of the full range. That meta-analysis also reports yield increases even without adaptation. This is not surprising in itself, since the models in the meta-analysis have their own shortcomings, and span a long history of research and a range of environments. Overall, this suggests medium agreement.

South Africa LT-RCP8.5 maize with incremental adaptation:

The mean percentage change to maize yield with autonomous adaptation for RCP8.5 is -5% (range across climate models -26 to 8%; 1/18 climate models are outliers). This becomes mean -4%, range -23 to 8% after removing the lower limit outliers. High confidence (medium robustness and high agreement).

Robustness assessment: Medium robustness. 0/18 climate models are outliers, which means highly robust with respect to climate model uncertainty; however, there is some uncertainty associated with crop model parameterisation (e.g. CO2 and response to duration, which differ across crop models; and only one crop model was run here). Therefore, the robustness assessment has been downgraded to medium.

Agreement assessment: High agreement. Many studies in the literature suggest that maize yields are likely to decline with climate change, even with some adaptation, including previously published meta-analysis showing very similar results with adaptation [5].

South Africa LT-RCP8.5 maize adaptation benefit:

The mean benefit of adaptation for maize for RCP8.5 is 38% of baseline yields (range across climate models 31 to 55%; 1/18 climate models are outliers).

This becomes mean 37%, range 31 to 52% after removing the upper limit outliers. Low confidence (medium robustness and low agreement).

Robustness assessment: Medium robustness. 1/18 climate models are outliers, which means high robustness with respect to climate model uncertainty; there is also some uncertainty associated with crop model parameterisation (e.g. CO2 and response to duration, which differ across crop models; and only one crop model was run here). Overall, this suggests medium robustness.

Agreement assessment: Low agreement. Many studies in the literature suggest that maize yields are likely to benefit from adaptation; there is uncertainty over how large this benefit is, although figures are typically much lower than those given by the iFEED projections [5], hence low agreement.

3 Future production implications - biodviersity, sustainability, costs, and crop pests and diseases (CPDs)

Under increasingly homogenised agricultural systems and reduced crop diversity, crop pest and disease pressures are expected to increase, with resultingly increased demands on chemical inputs and reduced crop yields. Under high production scenarios, there could be increased costs associated with increasing food production such as labour and agricultural input increases. Implication statements are used to summarise these risks, which are not present in the modelling framework. The calibrated statements (CS; concise model result summaries) are expanded upon by a wide range of academic experts who examine model results and provide implication statements (IS). This process builds upon the integrated modeling to ensure that processes/topics not explicitly modeled are included in final result summaries. IS are made in small academic expert teams around specific topic areas. These topic areas are agreed with in-country stakeholders and based on the expertise in the research team, selected to cover the various aspects of importance to climate change impacts and agricultural resilience in the region being studied. For full results, see ifeed.leeds.ac.uk.

The following implication statements give examples of these implications, taken from the Malawi HT-RCP8.5 scenario.

- There would be increased need for robust cross-sectoral planning and regulation for land and resources (e.g. water, energy, mining/minerals, biodiversity etc.).
- There would be increased need for infrastructural development, particularly for agricultural services e.g. storage, processing and transportation.
- Increased food production will require development of infrastructure for storage and processing to cut post-harvest losses and improve the value of agricultural commodities.

The following implication statements give examples of these implications, taken from the Tanzania HT-RCP8.5 scenario.

- Under such high climate risk and technological adoption scenarios, pest pressures are likely to increase due to climate change, especially in the highland farms; biocontrol by natural enemies will decline due to homogenisation and loss of biodiversity at the landscape and farm-scales affecting environmental sustainability and food system resilience.
- Homogenised agriculture focussing on fewer agricultural commodities reduce the inherent resilience and natural biocontrol of agricultural systems causing increased vulnerability to CPDs and associated crop damages and failures under climate risks.
- Climate change and increase in trade and market connectivity under homogenised agriculture facilitate the introduction and establishment of novel transboundary pests and diseases. Assuming that high technology adoption involves increase in globalised trade and market connectivity and causes expansion and intensification agriculture. Introduction and spread of novel transboundary pest and diseases depends on the crop protection measures employed by governments, as well as phytosanitary/biosecurity measures in place at points of entry for international imported commodities. Species-level information about pest responses are not clearly understood.
- Acute invasive species - such as fall army warm - could have a devastating impact on production leading to 60-100% of yield losses.
- Climate change and technological adoption will also reduce pressure from some pests, especially in lowland areas. Species-level information about pest responses are not clearly understood.
- Under high technology and climate risks, the focus will be on producing more food under challenging conditions while compromising environmental sustainability i.e., by using chemical fertilizers and pesticides and impacting

biodiversity, or conceding system resilience i.e., reduced local-scale crop diversity and increased dependence on international markets.

- Increased use of inputs (e.g. 'icides and fertilisers) would likely result in high-cost farming.

The following implication statements are taken from the Malawi LT-RCP2.6 scenario.

- Continued dominance of maize may contribute to low diet diversity - with detrimental health impacts expected, particularly for children, mothers and vulnerable and poor populations.
- With continued dominance of maize, we may expect minimal livelihood resilience, owing to less-diverse cropping opportunities (limiting risk spreading).
- With continued dominance of maize, we may expect minimal opportunities for on-farm income generation (e.g. market saturation with maize hence low prices).

4 Livestock expansion implications

The implications of expanding livestock production (a feature of all HT scenarios) include increasing land and resource conflicets, and increased agricultural emissions. The following implication statements give examples of these issues, taken from the Malawi HT-RCP8.5 scenario. For full results, see ifeed.leeds.ac.uk.

- Land conflicts expected to increase with expansion of agricultural land e.g. between livestock and crop production / between conservation and tourism, and between agricultural and energy (e.g. hydro).
- Water conflicts expected to increase between agricultural users and downstream consumers including transboundary escalation (e.g. Lower Shire River Basin and Zambezi river into Mozambique), including conflicts between livestock and crop production needs
- Increased livestock production would entail an increase in emissions and therefore measures would have to be taken across other sectors to offset the emissions and make the sector less carbon/emission intensive.
- Assumption of expansion of land use has huge implications for remaining Miombo woodlands and Ecosystem Service provision in Malawi [6].

5 iFEED in comparison to integrated assessment models

A range of models with differing degrees of complexity are available for assessing future food production and broader societal impacts of climate change [7]. Integrated Assessment Models (IAMs) describe interactions between the biophysical and socio-economic systems, providing projections of changes to land use and food production, and accounting for changes in dietary demand and costs of production. They depend upon partial or general equilibrium theory that accounts for supply and demand behaviours. General equilibrium models assess multiple interconnected markets; partial equilibirum models assess specific aspects of the economy whilst holding others constant. These approaches necessarily make several assumptions, such as that increased wealth leads to increased demand for livestock production in diets, as is seen in many current dietary trends. However, there are trends towards westernisation of diets in SSA, and in many western diets there are increasing trends towards plant-based products [8]. Therefore, the outcomes of these interrelated modelling processes are dependent on the partial or general equilibrium approaches inherent to them, and the assumptions that are necessary to drive these processes. Such models can therefore be difficult to interpret given the complexity of these interrelated processes [9, 10].

If models are too complex then the determination of, and interaction between, model parameters becomes difficult to constrain with observations [11]. On the other hand, if a model is too simple then important real-world processes are not captured. It can be said in general that there are multiple acceptable models, which may differ in their approach to appropriate complexity (the equifinaility thesis of Beven [12]). This thesis is especially relevant to food systems, where the processes simulated far exceed the data available for calibration and evaluation.

iFEED contrasts with IAMs in its approach to appropriate complexity. Regarding economics, rather than relying on complex partial or general equilibrium economic models, iFEED uses a qualitative international trade analysis that combines future projections of domestic food production with simple plausible scenarios of trade that bound future possibilities. Stakeholders directly inform the scenarios of trade that are of interest to them. This has the advantage of not having to rely on data for complex model evaluation, and has

transparency of inputs and output interpretation. Similarly, we do not seek to represent changing costs of production or changes to diets. Our focus instead is to model the biophysical, and rely on scenario analysis and stakeholder and expert interpretation of results to represent processes that are difficult to evaluate with confidence in models.

IAMs typically project future land use change in response to changing agricultural demand. For example, exploring scenarios of competing demands for urban, agricultural and protected land by mid-century [13], and linking West Africa-specific scenarios to global scenario analysis [14]. Both of these studies show the strengths of IAMs, quantifying how economics and agriculture might interact to deliver sufficient food by 2050. However, neither analysis provides country-specific scenarios, and changes in land use are ultimately derived from model projections more than stakeholder input. Another study [15] explores the benefits of using a combination of exploratory scenario analysis with normative backcasting, which attempts to backcast pathways towards desirable solutions, as envisaged by stakeholders. One of the conclusions of this study is that the whole process of scenario analysis should become increasingly owned by stakeholders.

Our approach is more directly driven by stakeholders than is typical in IAM scenario analysis. iFEED can explore the large-scale changes to land use and adaptation to climate change that stakeholders are interested in exploring. Our modelling system provides results showing how these changes can impact climate-smart nutrition security. We believe that both approaches (IAMs and iFEED) have strengths, and that a plurality of approaches is valuable for generating increasing confidence in evidence towards sensible pathways of change.

6 iFEED scoring system

The four components of climate smartness according to the Food and Agriculture Organisation are, to:

- Sustainably increase productivity
- Increase resilience through adaptation to climate change
- Reduce/remove GHGs (mitigation)
- Enhance achievement of national food (and nutrition) security and development goals

These four components encompass iFEED results. Here, we present a scoring system to evaluate each scenario in terms of each component of CSA, relative to the baseline. The negative / 0 / positive scoring detailed below is given to add detail to each aspect of the scoring system, with full results reported in Figure 1. For each component of CSA, the scoring system generally means:

- Blue: substantial improvement.
- Amber: improvement inconclusive; trade-offs to possible improvements.
- Red: clear inadequacy.

Note that a star indicates all aspects of productivity / adaptation / mitigation are improving or not worsening in that scenario; or for nutrition security, a star indicates all nutrient requirements are met for all trade vignettes

An overall summary score is given for each scenario, based on the following:

- Blue: more of the four criteria are improving than worsening.
- Amber: the same number of criteria are worsening as improving.
- Red: more of the four criteria are worsening than improving.

Productivity

The summary score for productivity is defined as follows:

- Blue: sustainable productivity increase and yield shocks not increasing.
- Amber: productivity increase, but questionable sustainability or yield shocks increasing.
- Red: productivity decreases or no significant change.

The following is used to define whether productivity changes, whether yield shocks change, and whether any increases to productivity are sustainable.

1a) Is productivity increased? If not, or no significant change, a negative score is given (Red). If it is then look at yield shocks (1b) and sustainability scores (1c) to determine Amber or Blue.

- 0 = no significant change (some climate model projections disagree on the sign of change).
- +/- = positive/negative change but <= 10% absolute change.
- ++/– > 10 < 100% absolute change.
- +++/— >= 100% absolute change.

1b). Are yield shock rates increasing? Note that no significant change in yield shocks is when some climate model projections disagree on the sign of change and the mean change across all climate models is less than 100%.

- If all / a majority / at least one crop shows signs of an increase in yield shocks and the other crops show no significant change: -
- If yield shock rates are not significantly changing for all crops: 0
- If yield shock rates are decreasing for all or a majority of crops, or if one shows a decrease and the others not a significant change: +

1c) Is the increase in productivity achieved at the expense of sustainability (environment, social, economic)? Specify those aspects potentially adversely impacted (e.g. yield increase through additional fertilizer likely to increase water (nitrate) and air (ammonia) pollution). For this, we require both of 1ci and 1cii to be + for the productivity increase to be considered sustainable:

i. Irrigation:

- 0 = no significant change (some climate model projections disagree on the sign of change).
- +/- = positive/negative change but $<=$ 10% absolute change.
- ++/– $>$ 10 $<$ 100% absolute change.
- +++/— $>=$ 100% absolute change.

ii. Other aspects of sustainability of the production system. Based on interpretation of the iFEED scenario summary documents:

- If changes to production system generally increase sustainability: +
- If no significant changes: 0
- If changes to production system generally decrease sustainability: -

Adaptation

The summary score for adaptation is defined as follows:

- Blue: adaptive capacity improving and both of SOC and community resilience are not worsening.

- Amber: adaptive capacity improving (2a), but one or both of SOC and community resilience are worsening.
- Red: adaptive capacity not improving.

The following is used to define whether adaptive capacity, SOC and community resilience changes.

2a) Is the production system likely to be more resilient in its adaptive capacity (crop varieties, irrigation, crop diversity)? Require a majority (or 1 improving and the other two no significant change) of the below to be positive for 2a to be positive: i. Irrigation change:

- 0 = no significant change (some climate model projections disagree on the sign of change)
- +/- = positive/negative change but <= 10% absolute change.
- ++/– >10 < 100% absolute change.
- +++/— >= 100% absolute change.

ii. Crop variety change:

- HT scenarios use duration-fix varieties where new varieties are assumed to be available, hence +.
- LT scenarios assume no new varieties, hence 0.

iii. Crop diversification change:

- +/- = positive/negative change but <= 10% change to maize area proportion.
- ++/– > 10 % change to maize area proportion, not including diversification even spread scenarios, or decrease in diversity reciprocal scenarios optimisation scenarios
- +++/— >= for optimisations where arable areas evenly spread, or the reciprocal optimisation decrease diversity scenarios.

2b) Are changes to soil organic carbon enhancing or reducing agricultural resilience?

- 0 = no significant change (some climate model projections disagree on the sign of change)

- +/- = positive/negative change but <= 10% absolute change.
- ++/– > 10 < 100% absolute change.
- +++/— >= 100% absolute change.

2c) Are communities likely to be more resilient? (e.g. does it improve the adaptive capacity of rural communities). Specify any groups within the community who are likely to be less resilient. Based on interpretation of the iFEED scenario summary documents:

- If changes to production system generally result in increased community resilience: +
- If no significant changes: 0
- If changes to production system generally result in decreased community resilience: -

Mitigation

The summary score for mitigation (reducing or removing GHG emissions and increasing SOC) is defined as follows:

- Blue: SOC increases and GHG emissions decrease; or one of these improve and the other does not significantly change
- Amber: one of SOC and GHG emissions improve and the other worsens; or both do not significantly change relative to the baseline
- Red: SOC decreases and GHG emissions increase; or one of these worsens and the other does not significantly change

The following is used to define whether SOC and GHG emissions change.

3a) Is more carbon stored in soil and/or vegetation (i.e. SOC change)?

- 0 = no significant change (some climate model projections disagree on the sign of change)
- +/- = positive/negative change but <= 10% absolute change.
- ++/– > 10 < 100% absolute change.
- +++/— >= 100% absolute change.

3b) Are GHG emissions reduced?

- 0 = no significant change (some climate model projections disagree on the sign of change)
- +/- = positive/negative change but <= 10% absolute change.
- ++/– > 10 < 100% absolute change.
- +++/— >=100% absolute change.

Nutrition security

The summary scorefor nutrition security is defined as follows.

- Blue: nutrient supply improves in all trade vignettes.
- Amber: trade vignettes disagree on improvement or no significant change or worsening.
- Red: nutrient supply worsens in all trade vignettes.

4a) Is nutrition security (i.e. adequate supply of nutrients at population level) improved for the population? This takes into account the self-sufficiency, BAU and stakeholder expectation (i.e. non-optimised) trade vignettes.

- Improvement (++ / +) / no significant change (0) / worsening (- /–) of nutrient supply for each trade vignette relative to baseline.
- NS indicates nutrient supply adequate at population level.
- ++ (strong improvement): If a majority of nutrients improve relative to the baseline AND more nutrient requirements are met relative to the baseline (more nutrients need to become adequate compared to the baseline than nutrients becoming inadequate compared to the baseline), nutrient supply is considered to improve strongly.
- + (improvement): If no additional nutrient requirements are fulfilled but the majority of nutrients improve relative to the baseline, nutrient supply is considered to improve.
- – (strong decline) If a majority of nutrients worsen relative to the baseline AND fewer nutrient requirements are met relative to the baseline (more nutrients need to become inadequate compared to the baseline than nutrients becoming adequate compared to the baseline), things are considered to strongly worsen.

- - (decline): If no additional nutrient requirements become inadequate but the majority of nutrients worsen relative to the baseline, nutrient supply is considered to worsen.
- 0 (no significant change): If things do not improve or worsen using the above criteria, nutrient supply is considered to not significantly change.
- If any of the 2050 projections overlap with the baseline (i.e. if some climate model projections disagree on the sign of change), then no significant change for that nutrient.
- All 2050 projections must have met the target when the baseline did not, or all 2050 projections should fail to meet the target when the baseline did, in order for a nutrient to be classed as becoming adequate or inadequate.

7 Supplementary figures and tables

Table 1 Source data for Figure 3a - per capita nutrient supplies with business-as-usual trade, relative to population requirements (100%) for HT-RCP2.6 in Tanzania. Baseline (2000) per capita nutrient levels, and five projected outcomes in 2050 under different climate models.

	Baseline	BCCCSM11	GFDLCM3	IPSLCM5ALR	MIROCESMCHEM	MRICGCM3
Dietary energy	112.00%	141.40%	145.40%	143.30%	137.90%	141.20%
Protein	161.00%	205.90%	214.90%	208.60%	197.50%	204.90%
Fat	68.30%	71.30%	71.90%	70.80%	71.10%	71.20%
Saturated fat	50.20%	52.20%	51.70%	51.50%	52.90%	52.20%
Vitamin C	262.10%	600.10%	623.40%	585.80%	564.30%	586.00%
Vitamin A	123.60%	235.70%	246.10%	232.20%	224.40%	232.30%
Folate	137.30%	192.70%	200.80%	191.70%	183.20%	191.10%
Calcium	36.60%	76.10%	79.20%	75.40%	72.30%	75.00%
Iron	58.50%	97.70%	101.60%	97.90%	92.60%	96.60%
Zinc	87.10%	122.20%	127.70%	123.90%	117.00%	121.70%
Dietary fibre	191.50%	294.90%	307.00%	295.80%	280.50%	292.40%
Thiamin	226.80%	496.40%	519.50%	497.20%	473.30%	492.20%
Riboflavin	156.30%	356.60%	367.40%	351.40%	339.10%	351.00%
Niacin	153.30%	238.30%	246.40%	240.10%	229.90%	236.90%
Vitamin B6	200.00%	340.20%	351.50%	338.90%	325.50%	336.50%

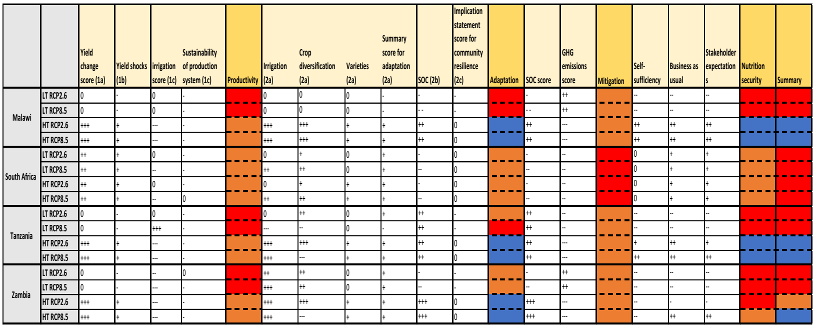

		Yield change score (1a)	Yield shocks (1b)	irrigation score (1c)	Sustainability of production system (1c)	Productivity	Irrigation (2a)	Crop diversification (2a)	Varieties (2a)	Summary score for adaptation (2a)	SOC (2b)	Implication statement score for community resilience (2c)	Adaptation	SOC score	GHG emissions score	Mitigation	Self-sufficiency	Business as usual	Stakeholder expectations	Nutrition security	Summary
Malawi	LT RCP2.6	0	-	0	-		0	0	0	-	-	-		-	++		--	--	--		
	LT RCP8.5	0	-	0	-		0	0	0	-	- -	-		- -	++		--	--	--		
	HT RCP2.6	+++	+	---	-		+++	+++	+	+	++	0		++	---		++	++	++		
	HT RCP8.5	+++	+	---	-		+++	+++	+	+	++	0		++	---		++	++	++		
South Africa	LT RCP2.6	++	+	0	-		0	+	0	+	-	0		-	--		0	+	+		
	LT RCP8.5	++	+	--	-		++	++	0	+	--	0		--	--		0	+	+		
	HT RCP2.6	++	+	0	-		0	+	+	+	-	0		-	--		0	+	+		
	HT RCP8.5	++	+	--	0		++	++	+	+	--	0		--	--		0	+	+		
Tanzania	LT RCP2.6	0	-	0	-		0	++	0	+	++	-		++	--		--	--	--		
	LT RCP8.5	0	-	+++	-		---	--	0	-	++	-		++	--		--	--	--		
	HT RCP2.6	+++	+	---	-		+++	+++	+	+	++	0		++	---		+	++	+		
	HT RCP8.5	+++	+	---	-		+++	---	+	+	++	0		++	---		++	++	++		
Zambia	LT RCP2.6	0	-	--	0		++	++	0	+	-	-		-	++		--	--	--		
	LT RCP8.5	0	-	---	-		+++	++	0	+	--	-		--	++		--	--	--		
	HT RCP2.6	+++	+	---	-		+++	+++	+	+	+++	0		+++	---		--	-	-		
	HT RCP8.5	+++	+	---	-		+++	---	+	+	+++	0		+++	---		--	++	++		

Fig. 1 iFEED results summary. LT = low transformation scenarios (low policy efficacy in Malawi; low market connectivity in Zambia; low technological development in Tanzania; low land reform in South Africa) and HT = high transformation. RCP2.6 = low climate risk. RCP8.5 = high climate risk. See Section 6 for full details of the scoring system.

Table 2 Source data for Figure 3b - per capita nutrient supplies with business-as-usual trade, relative to population requirements (100%) for HT-RCP8.5 in Tanzania. Baseline (2000) per capita nutrient levels, and five projected outcomes in 2050 under different climate models.

	Baseline	BCCCSM11	GFDLCM3	IPSLCM5ALR	MIROCESMCHEM	MRICGCM3
Dietary energy	112.00%	266.30%	271.60%	272.50%	272.40%	272.60%
Protein	161.00%	272.30%	278.60%	279.70%	281.70%	279.30%
Fat	68.30%	55.40%	54.80%	53.40%	52.50%	55.30%
Saturated fat	50.20%	51.40%	49.90%	46.90%	45.40%	50.50%
Vitamin C	262.10%	1164.60%	1184.00%	1205.60%	1300.70%	1158.30%
Vitamin A	123.60%	254.40%	258.40%	259.70%	277.00%	254.30%
Folate	137.30%	269.20%	274.10%	276.40%	291.80%	270.90%
Calcium	36.60%	88.10%	89.00%	89.40%	95.40%	87.90%
Iron	58.50%	136.80%	137.10%	138.70%	141.60%	139.40%
Zinc	87.10%	170.70%	175.40%	176.20%	176.20%	175.90%
Dietary fibre	191.50%	376.90%	384.20%	389.10%	395.20%	386.40%
Thiamin	226.80%	982.60%	1007.30%	1006.90%	1049.30%	992.20%
Riboflavin	156.30%	624.90%	623.50%	628.50%	665.30%	625.40%
Niacin	153.30%	323.70%	331.10%	333.60%	335.70%	332.60%
Vitamin B6	200.00%	517.60%	526.80%	531.40%	551.30%	525.40%

Table 3 Source data for Figure 4a - per capita nutrient supplies with business-as-usual trade, relative to population requirements (100%) for HT-RCP2.6 in Zambia. Baseline (2000) per capita nutrient levels, and five projected outcomes in 2050 under different climate models.

	Baseline	BCCCSM11	GFDLCM3	IPSLCM5ALR	MIROCESMCHEM	MRICGCM3
Dietary energy	100.70%	79.00%	77.30%	86.00%	79.40%	83.40%
Protein	159.00%	120.60%	118.20%	131.70%	120.80%	127.70%
Fat	63.60%	39.10%	38.10%	42.40%	39.00%	40.80%
Saturated fat	28.00%	15.30%	14.90%	16.40%	15.30%	15.80%
Vitamin C	209.60%	195.90%	188.00%	202.10%	199.70%	199.80%
Vitamin A	51.80%	50.70%	49.10%	53.30%	51.60%	51.90%
Folate	69.50%	60.30%	58.60%	64.10%	60.60%	62.60%
Calcium	21.90%	16.80%	16.40%	17.60%	16.90%	17.30%
Iron	64.50%	47.70%	46.70%	52.70%	47.60%	51.10%
Zinc	85.20%	67.80%	66.20%	74.00%	67.90%	71.80%
Dietary fibre	47.60%	39.30%	38.30%	42.50%	39.30%	41.50%
Thiamin	175.60%	169.40%	164.80%	184.60%	170.20%	179.20%
Riboflavin	90.20%	126.70%	122.80%	135.30%	130.30%	131.50%
Niacin	106.20%	99.10%	96.20%	106.70%	99.50%	104.00%
Vitamin B6	181.40%	262.00%	252.60%	279.30%	264.80%	272.70%

Table 4 Source data for Figure 4b - per capita nutrient supplies with business-as-usual trade, relative to population requirements (100%) for HT-RCP8.5 in Zambia. Baseline (2000) per capita nutrient levels, and five projected outcomes in 2050 under different climate models.

	Baseline	BCCCSM11	GFDLCM3	IPSLCM5ALR	MIROCESMCHEM	MRICGCM3
Dietary energy	100.70%	113.50%	119.90%	109.90%	105.30%	118.70%
Protein	159.00%	162.60%	171.80%	155.10%	149.00%	170.20%
Fat	63.60%	31.10%	31.90%	30.20%	31.00%	31.70%
Saturated fat	28.00%	12.50%	12.60%	12.30%	12.60%	12.60%
Vitamin C	209.60%	364.40%	369.70%	381.20%	334.80%	368.10%
Vitamin A	51.80%	56.90%	59.60%	58.20%	54.90%	59.00%
Folate	69.50%	93.60%	97.00%	93.30%	86.60%	96.40%
Calcium	21.90%	26.20%	26.70%	26.00%	24.60%	26.60%
Iron	64.50%	69.40%	73.90%	66.40%	63.20%	73.20%
Zinc	85.20%	94.10%	99.60%	90.40%	86.00%	98.60%
Dietary fibre	47.60%	60.20%	63.50%	58.50%	56.40%	62.90%
Thiamin	175.60%	269.10%	283.70%	264.00%	245.30%	281.10%
Riboflavin	90.20%	204.90%	219.20%	214.30%	201.60%	216.80%
Niacin	106.20%	128.40%	133.00%	124.40%	113.60%	132.40%
Vitamin B6	181.40%	453.00%	472.90%	460.40%	414.80%	469.10%

Table 5 Percentage Reference Nutrient Intake (RNI) values for the baseline, and 2050, assuming all maize and sugarcane as in the HT RCP8.5 scenario in Tanzania, as well as various scenarios of reduced maize and sugarcane in 2050. 2050A = 100% maize, 100% sugarcane, 2050B = 100% maize, no sugarcane, 2050C = no maize, 100% sugarcane, 2050D = 50% maize, 100% sugarcane, 2050E = 50% maize, no sugarcane. This supplementary analysis for Tanzania shows that reducing maize and sugarcane supply in the HT-RCP8.5 scenario still allows nutrients to improve, meaning that overconsumption of these crops is not necessary for improved nutrition outcomes. However, excessive production of these crops could encourage overconsumption of less micronutrient-rich foods, which could have consequences for increasing obesity, as well as negative environmental impacts from sub-optimal agricultural expansion.

	Baseline	2050A	2050B	2050C	2050D	2050E
Food (calories / energy)	112	226	187	130	178	139
Protein	161	270	270	147	209	209
Fat	68	60	60	40	50	50
Saturated Fat	50	52	52	46	49	49
Vitamin C	262	1167	1167	1167	1167	1167
Vitamin A (RAE)	124	250	250	250	250	250
Folate	137	269	269	226	248	248
Calcium	37	88	87	84	86	86
Iron	58	135	134	71	103	102
Zinc	87	169	169	81	125	125
Dietary Fibre	191	374	374	203	289	289
Thiamin	220	980	980	750	865	865
Riboflavin	150	618	614	504	561	557
Niacin	153	321	321	160	241	241
Vitamin B6	191	516	516	359	438	438

Table 6 Size and composition of iFEED stakeholder taskforce for each focal country. Rows with repeated organisations indicate multiple taskforce members from that organisation.

Malawi (17 members)	South Africa (12 members)	Tanzania (13 members)	Zambia (7 members)
LUANAR	ARC	Ministry of Agriculture	
LUANAR	ARC	Ministry of Agriculture	National Project Coordinating Unit (PPCR Zambia & TRALARD)
National Planning Commission	DAFF	Presidents Office (PORALG)	
National Statistical Office	Potato SA	National Land Use Planning Commission	Food and Agriculture Organization of the United Nations (FAO)
Mwapata Institute	SANTAM	Vice presidents Office Environment Division	International Development Enterprise (IDE)
Concern Worldwide	Provincial Agriculture Free State	Sokoine University of Agriculture	Indaba Agricultural Policy Research Institute (IAPRI)
Care Malawi	Provincial Agriculture Free State	National Irrigation Commission	Ministry of Agriculture
NASFAM	AFASA Women FS	Tanzania Food and Nutrition Centre	
Chancellor College	AFASA Women NW	Ministry of Livestock and Fisheries	
Trocaire	University of Free State Agric Eco	Tanzania CSA Association	
Total Land Care	Agrometeorologys	Tanzania Meteorological Agency (TMA)	
Save the Children	GM Research and Intelligence	Tanzania Agriculture Research Institute	
Welthungerhilfe		National Bureau of Statistics	
Farmers Union			
Ministry of Disaster Management			
Ministry of Disaster Management			
CISANET Board Chairperson / AICC			

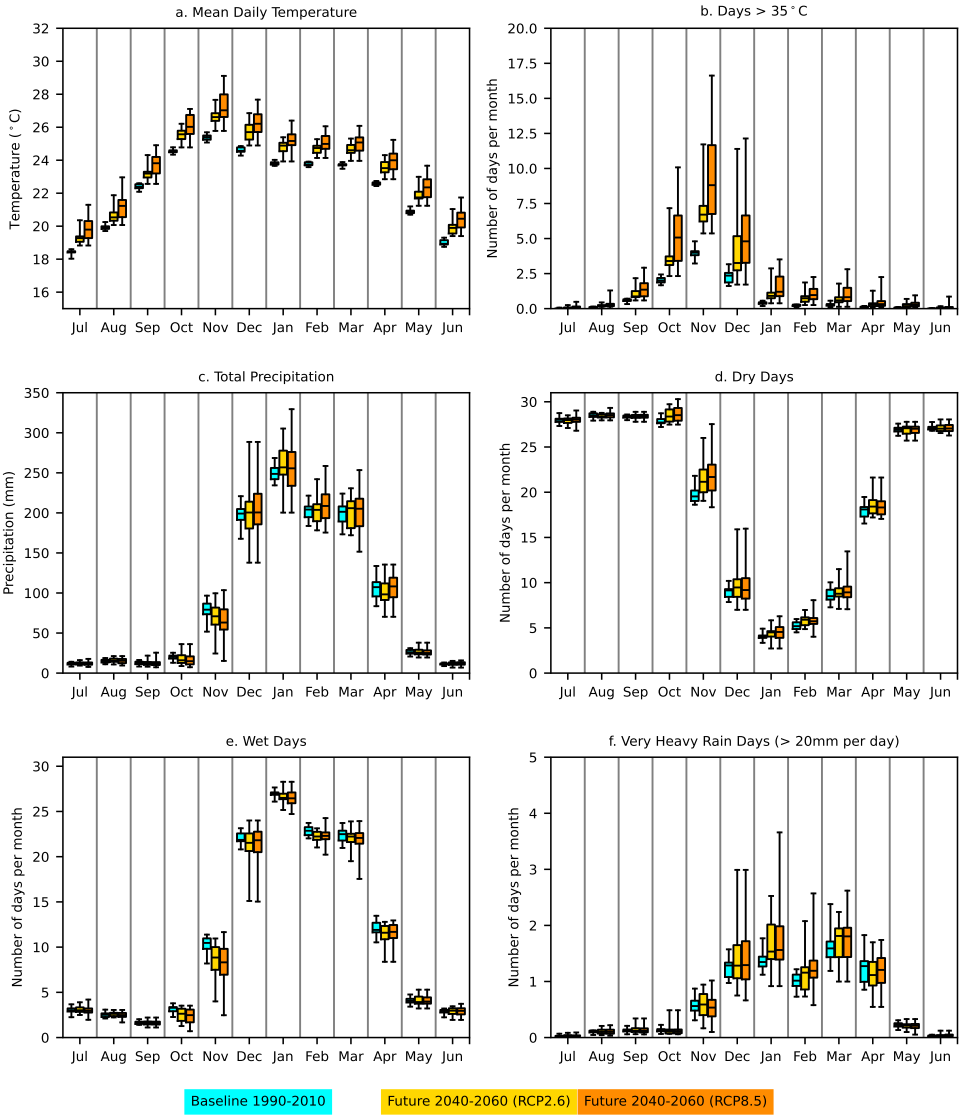

Fig. 2 Climate indices for Malawi. a. Mean Daily Temperature, b. Days > 35°C, c. Total Precipitation, d. Dry Days, e. Wet Days, and f. Very Heavy Rain Days (> 20 mm per day). Data shows the baseline (1990 to 2010; cyan) and future (2040 to 2060) conditions for both low (RCP2.6; yellow) and high (RCP8.5; orange) climate risk scenarios. Horizontal lines in the boxes show the median, the box boundaries show the 25th to 75th percentile ranges and the whiskers show the minima and maxima of the data (n = 18).

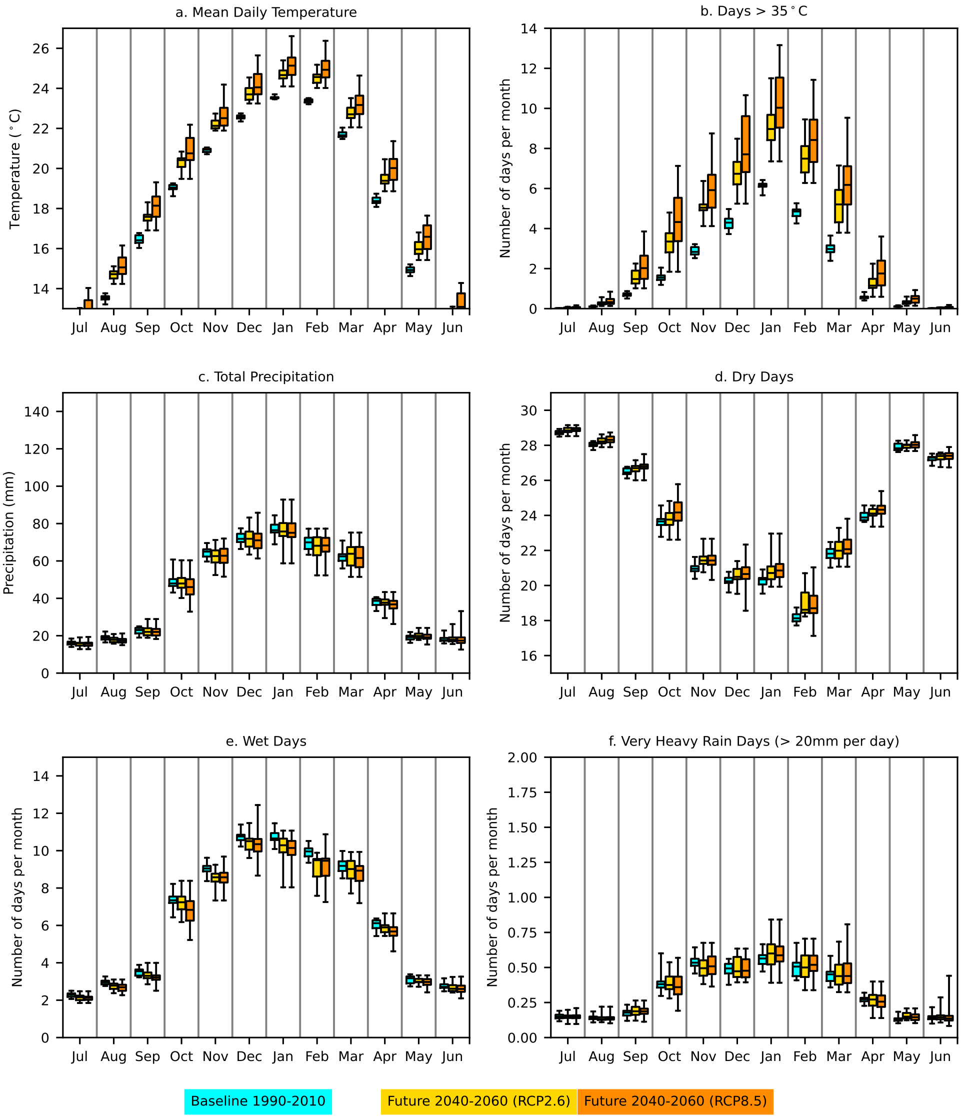

Fig. 3 Climate indices for South Africa. a. Mean Daily Temperature, b. Days > 35°C, c. Total Precipitation, d. Dry Days, e. Wet Days, and f. Very Heavy Rain Days (> 20 mm per day). Data shows the baseline (1990 to 2010; cyan) and future (2040 to 2060) conditions for both low (RCP2.6; yellow) and high (RCP8.5; orange) climate risk scenarios. Horizontal lines in the boxes show the median, the box boundaries show the 25th to 75th percentile ranges and the whiskers show the minima and maxima of the data (n = 18).

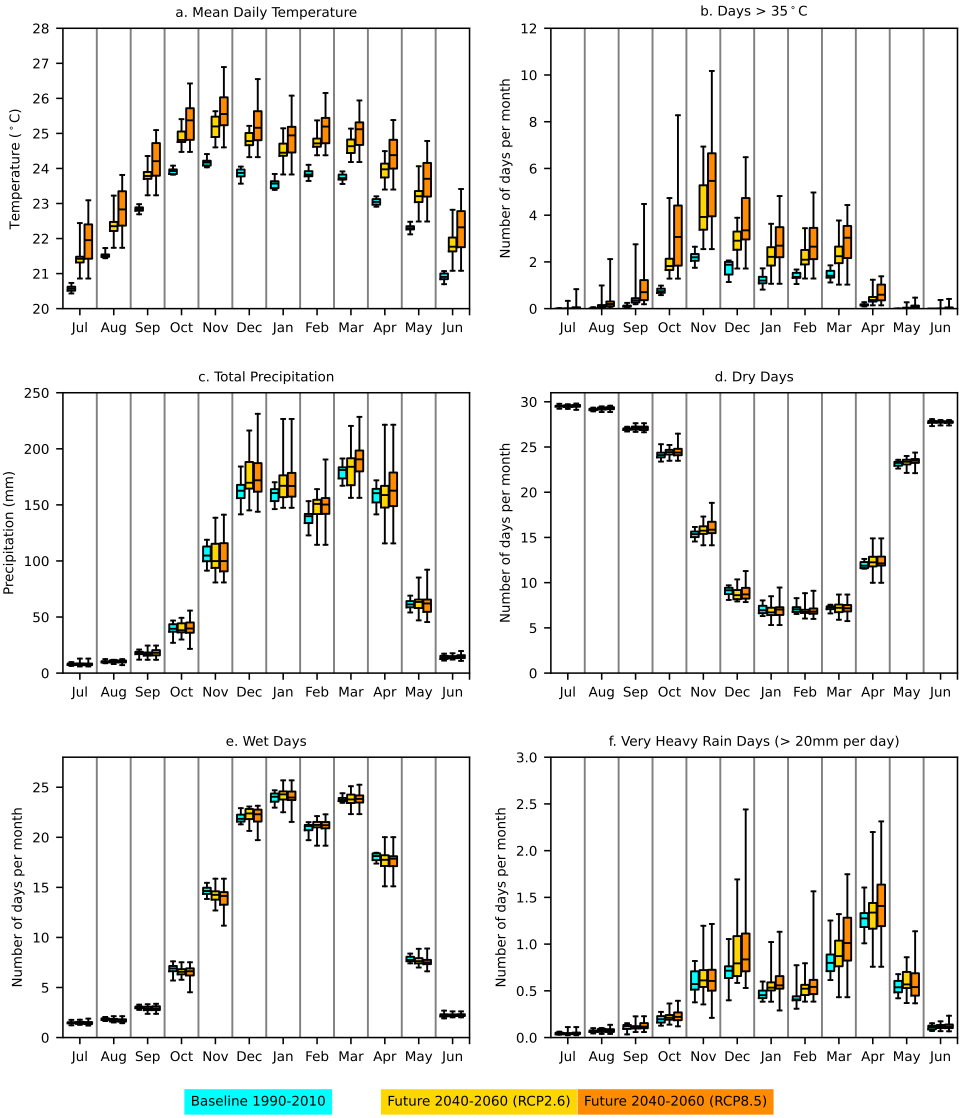

Fig. 4 Climate indices for Tanzania. a. Mean Daily Temperature, b. Days > 35°C, c. Total Precipitation, d. Dry Days, e. Wet Days, and f. Very Heavy Rain Days (> 20 mm per day). Data shows the baseline (1990 to 2010; cyan) and future (2040 to 2060) conditions for both low (RCP2.6; yellow) and high (RCP8.5; orange) climate risk scenarios. Horizontal lines in the boxes show the median, the box boundaries show the 25th to 75th percentile ranges and the whiskers show the minima and maxima of the data (n = 18).

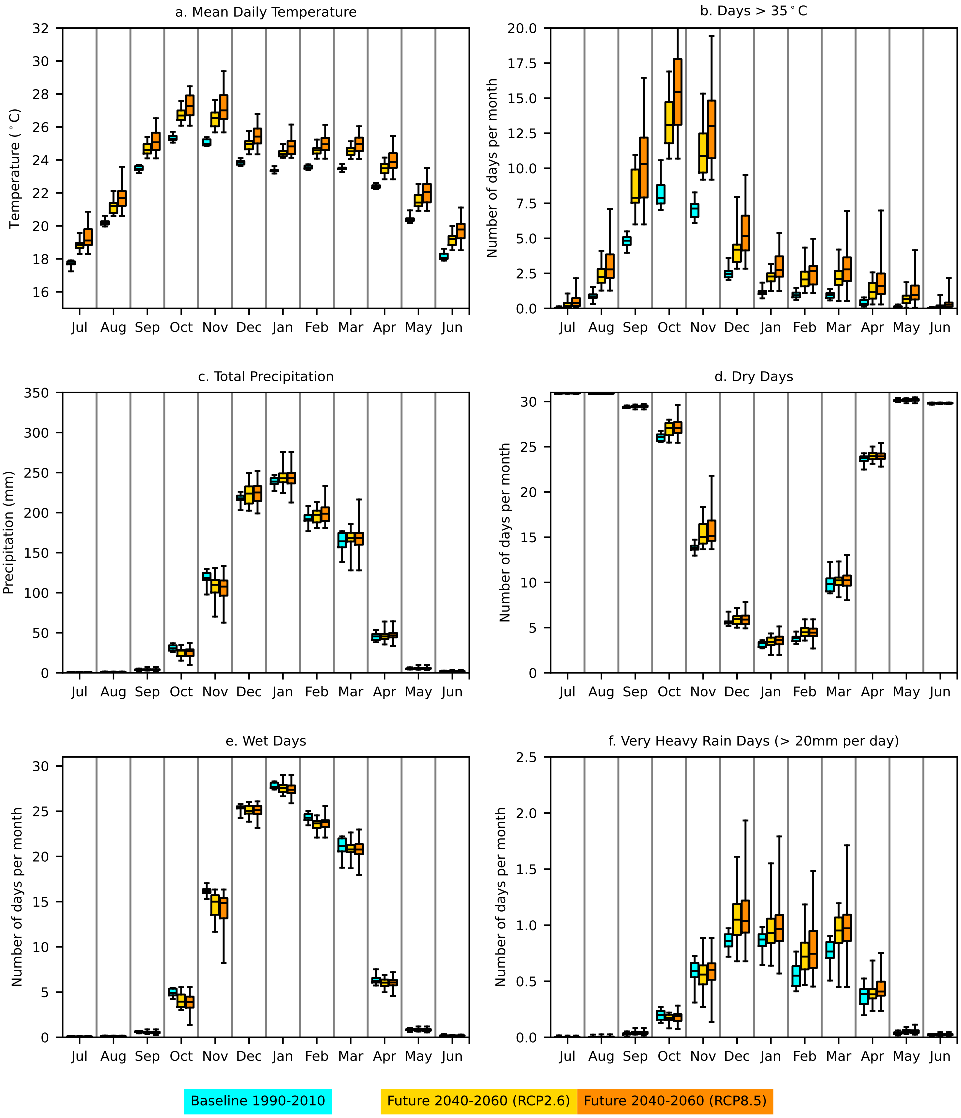

Fig. 5 Climate indices for Zambia. a. Mean Daily Temperature, b. Days > 35°C, c. Total Precipitation, d. Dry Days, e. Wet Days, and f. Very Heavy Rain Days (> 20 mm per day). Data shows the baseline (1990 to 2010; cyan) and future (2040 to 2060) conditions for both low (RCP2.6; yellow) and high (RCP8.5; orange) climate risk scenarios. Horizontal lines in the boxes show the median, the box boundaries show the 25th to 75th percentile ranges and the whiskers show the minima and maxima of the data (n = 18).

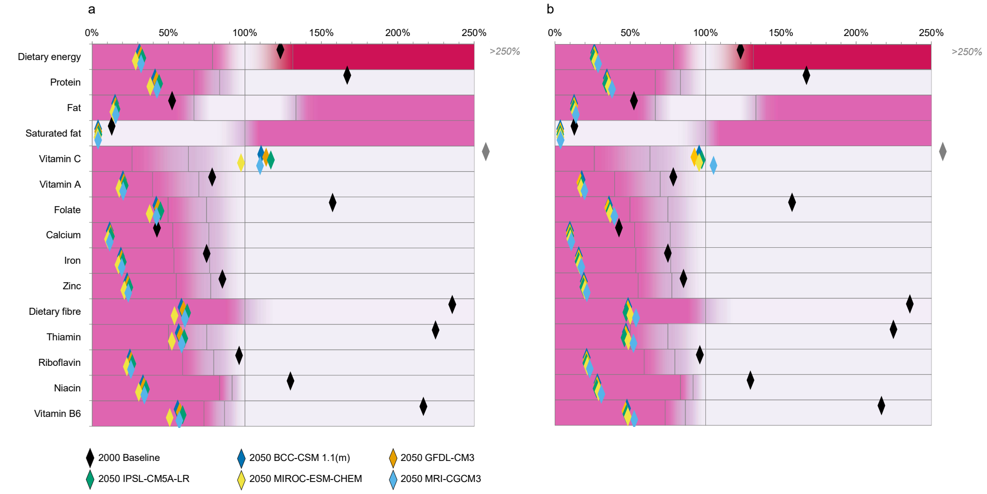

Fig. 6 Per capita nutrient supplies with business-as-usual trade, relative to population requirements (100%) for a). LT-RCP2.6 and b). LT-RCP8.5 in Malawi. Black diamonds indicate baseline (2000) per capita nutrient levels. The five coloured diamonds indicate the projected outcomes in 2050 under different climate models. Grey areas indicate where per capita nutrient requirements are met and pink areas indicate that requirement are not achieved, with intermediate areas marginal. For all nutrients other than energy and fat, the first threshold represents the Lower Reference Nutrient Intake (LRNI); the second, the Estimated Average Requirement (EAR); the third, the Reference Nutrient Intake (RNI; principal target). For fat, thresholds correspond to minimum, min-max midpoint, and maximum recommended intakes respectively. For energy, the respective thresholds are MDER, ADER, and XDER (minimum, average and maximum dietary energy requirements). The dark pink area indicates where calories are greater than requirements. Vitamin A is measured in retinol activity equivalents (RAE).

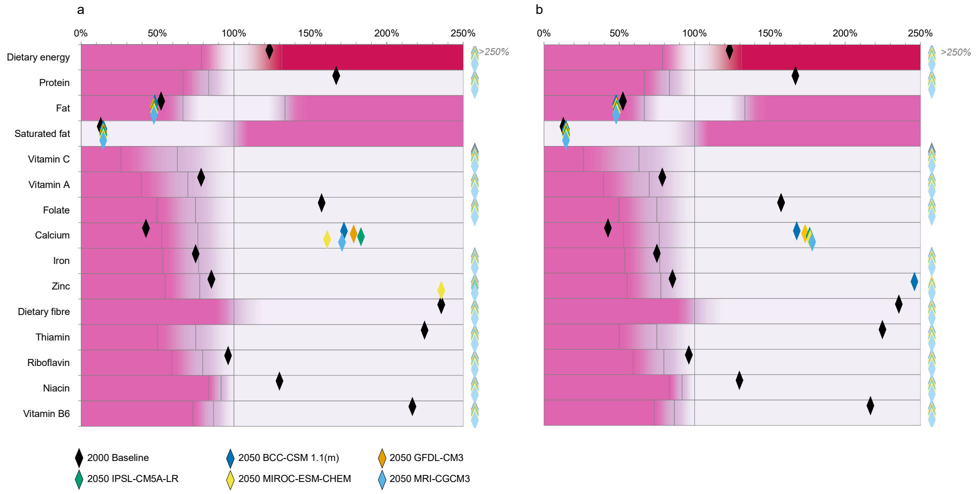

Fig. 7 Per capita nutrient supplies with business-as-usual trade, relative to population requirements (100%) for a). HT-RCP2.6 and b). HT-RCP8.5 in Malawi. Black diamonds indicate baseline (2000) per capita nutrient levels. The five coloured diamonds indicate the projected outcomes in 2050 under different climate models. Grey areas indicate where per capita nutrient requirements are met and pink areas indicate that requirement are not achieved, with intermediate areas marginal. For all nutrients other than energy and fat, the first threshold represents the Lower Reference Nutrient Intake (LRNI); the second, the Estimated Average Requirement (EAR); the third, the Reference Nutrient Intake (RNI; principal target). For fat, thresholds correspond to minimum, min-max midpoint, and maximum recommended intakes respectively. For energy, the respective thresholds are MDER, ADER, and XDER (minimum, average and maximum dietary energy requirements). The dark pink area indicates where calories are greater than requirements. Vitamin A is measured in retinol activity equivalents (RAE).

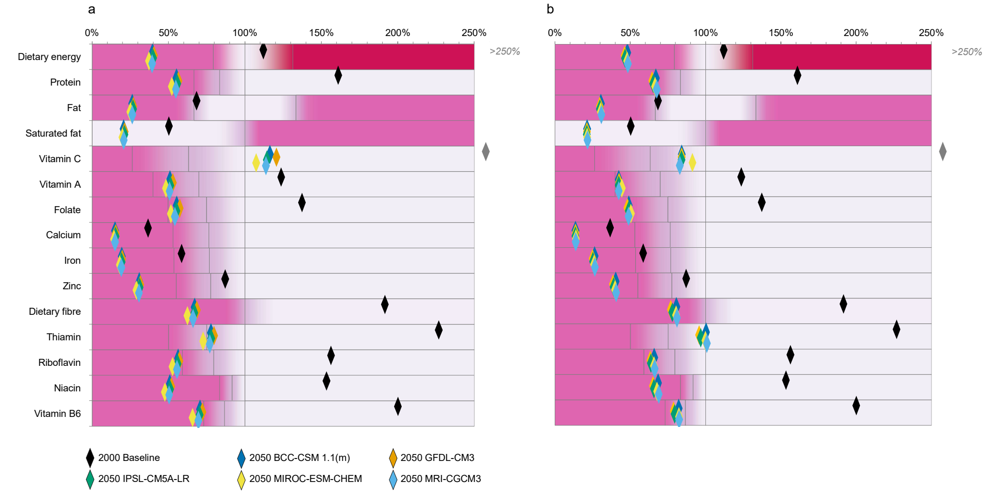

Fig. 8 Per capita nutrient supplies with business-as-usual trade, relative to population requirements (100%) for a). LT-RCP2.6 and b). LT-RCP8.5 in Tanzania. Black diamonds indicate baseline (2000) per capita nutrient levels. The five coloured diamonds indicate the projected outcomes in 2050 under different climate models. Grey areas indicate where per capita nutrient requirements are met and pink areas indicate that requirement are not achieved, with intermediate areas marginal. For all nutrients other than energy and fat, the first threshold represents the Lower Reference Nutrient Intake (LRNI); the second, the Estimated Average Requirement (EAR); the third, the Reference Nutrient Intake (RNI; principal target). For fat, thresholds correspond to minimum, min-max midpoint, and maximum recommended intakes respectively. For energy, the respective thresholds are MDER, ADER, and XDER (minimum, average and maximum dietary energy requirements). The dark pink area indicates where calories are greater than requirements. Vitamin A is measured in retinol activity equivalents (RAE).

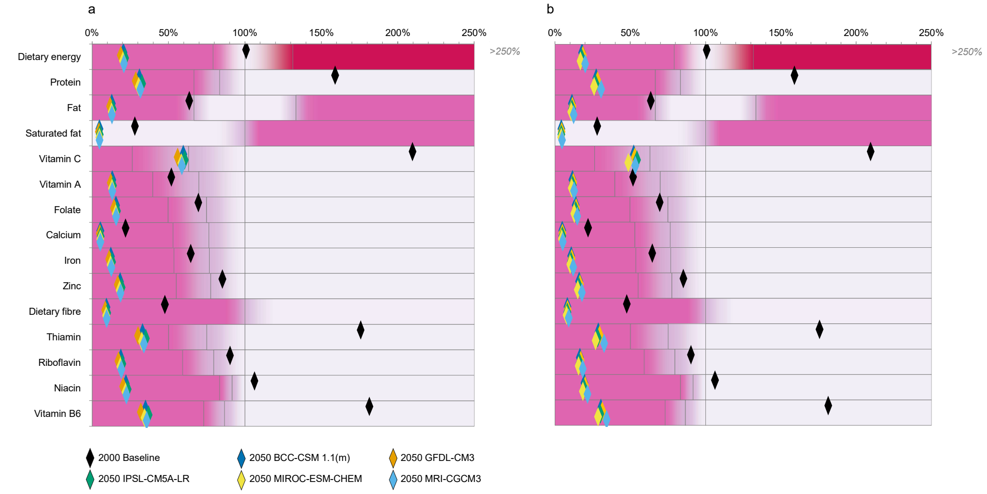

Fig. 9 Per capita nutrient supplies with business-as-usual trade, relative to population requirements (100%) for a). LT-RCP2.6 and b). LT-RCP8.5 in Zambia. Black diamonds indicate baseline (2000) per capita nutrient levels. The five coloured diamonds indicate the projected outcomes in 2050 under different climate models. Grey areas indicate where per capita nutrient requirements are met and pink areas indicate that requirement are not achieved, with intermediate areas marginal. For all nutrients other than energy and fat, the first threshold represents the Lower Reference Nutrient Intake (LRNI); the second, the Estimated Average Requirement (EAR); the third, the Reference Nutrient Intake (RNI; principal target). For fat, thresholds correspond to minimum, min-max midpoint, and maximum recommended intakes respectively. For energy, the respective thresholds are MDER, ADER, and XDER (minimum, average and maximum dietary energy requirements). The dark pink area indicates where calories are greater than requirements. Vitamin A is measured in retinol activity equivalents (RAE).

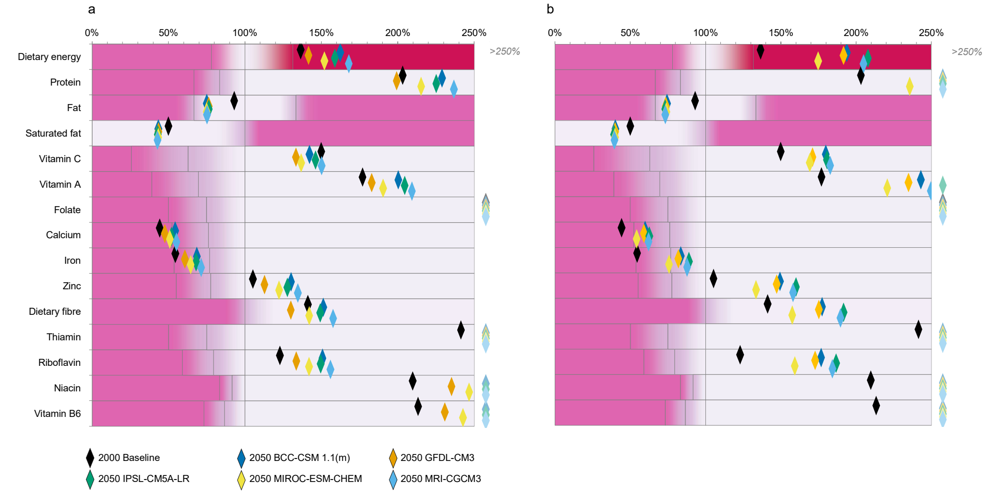

Fig. 10 Per capita nutrient supplies with business-as-usual trade, relative to population requirements (100%) for a). LT-RCP2.6 and b). LT-RCP8.5 in South Africa. Black diamonds indicate baseline (2000) per capita nutrient levels. The five coloured diamonds indicate the projected outcomes in 2050 under different climate models. Grey areas indicate where per capita nutrient requirements are met and pink areas indicate that requirement are not achieved, with intermediate areas marginal. For all nutrients other than energy and fat, the first threshold represents the Lower Reference Nutrient Intake (LRNI); the second, the Estimated Average Requirement (EAR); the third, the Reference Nutrient Intake (RNI; principal target). For fat, thresholds correspond to minimum, min-max midpoint, and maximum recommended intakes respectively. For energy, the respective thresholds are MDER, ADER, and XDER (minimum, average and maximum dietary energy requirements). The dark pink area indicates where calories are greater than requirements. Vitamin A is measured in retinol activity equivalents (RAE).

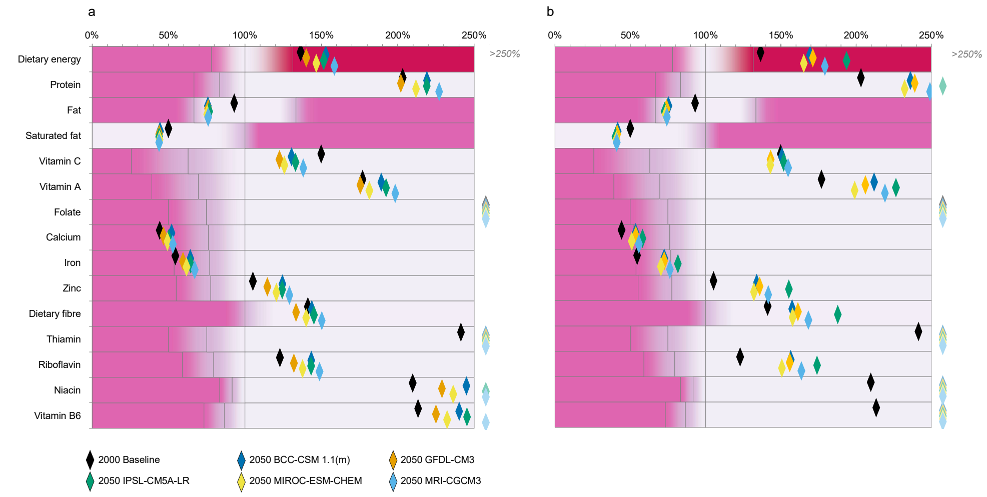

Fig. 11 Per capita nutrient supplies with business-as-usual trade, relative to population requirements (100%) for a). HT-RCP2.6 and b). HT-RCP8.5 in South Africa. Black diamonds indicate baseline (2000) per capita nutrient levels. The five coloured diamonds indicate the projected outcomes in 2050 under different climate models. Grey areas indicate where per capita nutrient requirements are met and pink areas indicate that requirement are not achieved, with intermediate areas marginal. For all nutrients other than energy and fat, the first threshold represents the Lower Reference Nutrient Intake (LRNI); the second, the Estimated Average Requirement (EAR); the third, the Reference Nutrient Intake (RNI; principal target). For fat, thresholds correspond to minimum, min-max midpoint, and maximum recommended intakes respectively. For energy, the respective thresholds are MDER, ADER, and XDER (minimum, average and maximum dietary energy requirements). The dark pink area indicates where calories are greater than requirements. Vitamin A is measured in retinol activity equivalents (RAE).

References

[1] Bradshaw, C.D., Pope, E., Kay, G., Davie, J.C., Cottrell, A., Bacon, J., Cosse, A., Dunstone, N., Jennings, S., Challinor, A., *et al.*: Unprecedented climate extremes in South Africa and implications for maize production. Environmental Research Letters **17**(8), 084028 (2022)

[2] Thomas, T.S., Robertson, R.D., Strzepek, K., Arndt, C.: Extreme events and production shocks for key crops in southern africa under climate change. Frontiers in Climate **4** (2022). https://doi.org/10.3389/fclim.2022.787582

[3] Jennings, S.A., Challinor, A.J., Smith, P., Macdiarmid, J., Pope, E., Chapman, S., Bradshaw, C., Clark, H., Vetter, S., Fitton, N., King, R., Mwamakamba, S., Madzivhandila, T., Mashingaidze, I., Chomba, C., Nawiko, M., Nyhodo, B., Mazibuko, N., Yeki, P., Kuwali, P., Kambwiri, A., Kazi, V., Kiama, A., Songole, A., Coskeran, H., Quinn, C., Sallu, S., Dougill, A., Whitfield, S., Kunin, B., Meebelo, N., Jamali, A., Kantande, D., Makundi, P., Mbungu, W., Kayula, F., Walker, S., Zimba, S., Yamdeu, J.H.G., Kapulu, N., Galdos, M.V., Eze, S., Tripathi, H., Sait, S.M., Kepinski, S., Likoya, E., Greathead, H., Smith, H.E., Mahop, M.T., Harwatt, H., Muzammil, M., Horgan, G., Benton, T.: A new integrated assessment framework for climate-smart nutrition security in sub-Saharan Africa: the integrated Future Estimator for Emissions and Diets (iFEED). Frontiers in Sustainable Food Systems (2022)

[4] Parkes, B., Challinor, A., Nicklin, K.: Crop failure rates in a geoengineered climate: impact of climate change and marine cloud brightening. Environmental Research Letters **10**(8), 084003 (2015)

[5] Challinor, A.J., Watson, J., Lobell, D.B., Howden, S.M., Smith, D.R., Chhetri, N.: A meta-analysis of crop yield under climate change and adaptation. Nature Climate Change (4), 1–5 (2014). https://doi.org/10.1038/NCLIMATE2153

[6] Ryan, C.M., Pritchard, R., McNicol, I., Owen, M., Fisher, J.A., Lehmann, C.: Ecosystem services from southern African woodlands and their future under global change. Philosophical Transactions of the Royal Society B: Biological Sciences **371**(1703), 20150312 (2016)

[7] Challinor, A.J., Osborne, T., Shaffrey, L., Weller, H., Morse, A., Wheeler, T., Vidale, P.L.: Methods and Resources for Climate Impacts Research. Bulletin of the American Meteorological Society **90**(6), 836–848 (2009). https://doi.org/10.1175/2008BAMS2403.1

[8] Wickramasinghe, K., Breda, J., Berdzuli, N., Rippin, H., Farrand, C., Halloran, A.: The shift to plant-based diets: are we missing the point? Global Food Security **29**, 100530 (2021)

[9] Webber, H., Gaiser, T., Ewert, F.: What role can crop models play in supporting climate change adaptation decisions to enhance food security in sub-saharan africa? Agricultural Systems **127**, 161–177 (2014). https://doi.org/10.1016/j.agsy.2013.12.006. Times Cited: 1 1

[10] Ewert, F., Rötter, R.P., Bindi, M., Webber, H., Trnka, M., Kersebaum, K.C., Olesen, J.E., van Ittersum, M.K., Janssen, S., Rivington, M., *et al.*: Crop modelling for integrated assessment of risk to food production from climate change. Environmental Modelling & Software **72**, 287–303 (2015)

[11] Challinor, A., Muller, C., Asseng, S., Deva, C., Nicklin, K., Wallach, D., Vanuytrecht, E., Whitfield, S., Ramirez-Villegas, J., Koehler, A.: Improving the use of crop models for risk assessment and climate change adaptation. Agricultural Systems **159**, 296–306 (2018)

[12] Beven, K.: A manifesto for the equifinality thesis. Journal of Hydrology **320**(1-2), 18–36 (2006)

[13] van Soesbergen, A., Arnell, A.P., Sassen, M., Stuch, B., Schaldach, R., Göpel, J., Vervoort, J., Mason-DCroz, D., Islam, S., Palazzo, A.: Exploring future agricultural development and biodiversity in Uganda, Rwanda and Burundi: a spatially explicit scenario-based assessment. Regional Environmental Change **17**, 1409–1420 (2017)

[14] Palazzo, A., Vervoort, J.M., Mason-DCroz, D., Rutting, L., Havlík, P., Islam, S., Bayala, J., Valin, H., Kadi, H.A.K., Thornton, P., *et al.*: Linking regional stakeholder scenarios and shared socioeconomic pathways: Quantified West African food and climate futures in a global context. Global Environmental Change **45**, 227–242 (2017)

[15] Vervoort, J.M., Thornton, P.K., Kristjanson, P., Förch, W., Ericksen, P.J., Kok, K., Ingram, J.S., Herrero, M., Palazzo, A., Helfgott, A.E., *et al.*: Challenges to scenario-guided adaptive action on food security under climate change. Global Environmental Change **28**, 383–394 (2014)
